# Architectural patterns for health information systems: a systematic review

**DOI:** 10.3389/fdgth.2025.1694839

**Published:** 2025-11-17

**Authors:** Rene Casanova, Fernan A. Villa-Garzon, John W. Branch-Bedoya

**Affiliations:** Departamento de Ciencias de la Computación y la Decisión, Universidad Nacional de Colombia, Medellín, Colombia

**Keywords:** digital health ecosystems, software architecture, interoperability, microservices, blockchain, HL7 FHIR, systematic review

## Abstract

**Background:**

Health information systems (HIS) are critical for digital health transformation, yet fragmentation and poor interoperability adoption remains a major challenge.

**Objectives:**

This study systematically reviews architectural patterns used in HIS and evaluates their alignment with ecosystem-level requirements.

**Methods:**

Following PRISMA 2020 guidelines, a systematic literature review was conducted across Scopus, IEEE Xplore, PubMed, and Web of Science (2020–2025). Eligible studies described, evaluated, or proposed HIS solutions.

**Results:**

From an initial set of 304 records, 89 met the inclusion criteria. Service-based and decentralized/distributed ledger architectures were predominant, with emerging models integrating edge computing and modular design. FHIR-based contracts are found as stabilizers of interfaces, enabling validation and reducing integration costs. However, gaps persist in cross-border care, sustainability, and artificial intelligence integration.

**Conclusion:**

While microservices dominate current HIS architectures, achieving resilient, interoperable ecosystems requires greater architectural diversity and intersectoral collaboration.

## 1 Introduction

Digital health ecosystems rely on health information systems (HIS) as foundational components for delivering integrated and patient-centered care. These systems enable the capture, storage, and exchange of clinical and administrative data, which is essential for maintaining continuity of care, planning resources, and managing population health. However, their design and implementation must account for the inherent complexity of healthcare environments, which involve multiple stakeholders, heterogeneous processes, and dynamic regulatory frameworks.

Despite technological advances, healthcare ecosystems often function as collections of isolated and heterogeneous software solutions (1), lacking standardized integration frameworks. This fragmentation undermines interoperability and complicates efforts to establish sustainable, modular, and scalable digital health infrastructures. Over the past decade, software engineering has introduced multiple architectural strategies aiming at improving interoperability and resilience.

Among these, service-oriented and microservice architectures enhance modularity and deployability by decomposing systems into fine-grained, independently manageable components. Distributed and decentralized ledger-oriented architectures address data exchange trust, auditability, and security through shared and immutable transaction records. Layered architectures remain prevalent because of their capacity to separate concerns and facilitate maintainability. Edge-cloud computing architectures leverage distributed processing by bringing computation closer to data sources while ensuring elasticity and scalability through cloud integration. Process-driven and pipeline architectures support business intelligence and analytics workflows, improving transparency, adaptability, and workflow automation. Finally, machine learning and artificial intelligence integration architectures enable predictive modeling and adaptive decision support directly within clinical and administrative processes.

From an end-user perspective, the value of these architectural strategies lies in their ability to transform data into real-world actionable insights. Reliable architectures ensure that collected information remains accurate, complete, and timely, enabling consumers to make informed clinical and managerial decisions. Moreover, the quality of data generated and exchanged between systems directly influences the scope and reliability of artificial intelligence applications (2, 3), which depend on consistent, high-quality datasets for training and inference. By improving data integrity, well-designed architectures ultimately enhance the usability, trustworthiness, and impact of digital health tools throughout the continuum of healthcare provisioning.

Nevertheless, the adoption of best practices in real-world healthcare contexts remains inconsistent, and the extent to which these approaches support ecosystem-level principles such as openness, sustainability, and cross-border integration remains unclear. Furthermore, while reference models such as HL7 and open EHR have emerged as interoperability enablers, there is limited evidence on how these standards are combined with architectural strategies in complete production environments. A critical knowledge gap persists regarding which patterns dominate current HIS designs, which ones are emerging, and whether these architectures align with the requirements of complex digital health ecosystems, such as those conceptualized under Software Ecosystem (SECO) and System-of-Systems (SoS) paradigms.

To address this gap, we conducted a systematic literature review (SLR) following PRISMA 2020 guidelines, analyzing studies published between 2020 and 2025. The objective was to identify predominant architectural patterns in HIS, assess their application contexts, and evaluate their alignment with ecosystem-level requirements for digital health. This synthesis aims to inform researchers and practitioners about current trends and future directions for designing interoperable, resilient, and sustainable health information systems.

## 2 Methods

### 2.1 Protocol and registration

This study was carried out in accordance with the Preferred Reporting Items for Systematic Reviews and Meta-Analysis (PRISMA) 2020 guidelines (4) to ensure transparency and reproducibility. The review protocol was not registered in PROSPERO or similar registries due to its exploratory scope. A complete PRISMA 2020 checklist is provided in the Supplementary Material, and the flow diagram is depicted in Figure 2.

### 2.2 Information sources

Four major electronic databases were queried. The selected information sources are described in Table 1.

**Table 1:** Data sources included in this review.

Data source	Type	URL	Last update
Scopus	Database	1	14 June 2025
PubMed	Archive	2	18 June 2025
IEEE Xplore	Digital library	3	22 June 2025
Web of Science	Database	4	20 June 2025

The last search was conducted on 22 June 2025. The databases were selected for their broad coverage of healthcare and information technology literature. The exported files are available at the project repository.

### 2.3 Search strategy

Search strategies were designed to comprehensively capture studies related to *health information systems* and *software architecture*. Keyword combinations were constructed using Boolean operators and adapted to the syntax of each database. Searches were limited to the period January 2020 to June 2025 and targeted articles describing, evaluating, or proposing architectural models for health information systems.

The structure of the search strings combined controlled vocabulary (e.g., MeSH terms in PubMed) and free-text terms referring to “health information systems,” “architecture,” “software design,” and “interoperability.” Equivalent terms were harmonized across databases to ensure semantic consistency.

The following is the summary of search parameters:

- **Databases queried:**
  ○ Scopus
  ○ PubMed
  ○ IEEE Xplore
  ○ Web of Science
- **Domains:** Health information systems, software architecture, digital health ecosystems
- **Time frame:** 2020–2025
- **Languages:** English, Portuguese, and Spanish
- **Last search update:** 22 June 2025

The complete search strings and query syntax for each database are provided in the Supplementary Material.

### 2.4 Eligibility criteria

Elegibility criteria were defined to ensure methodological rigor and maintain alignment between the research objectives and the evidence included in this systematic review. Following PRISMA 2020 recommendations, criteria were applied in two sequential stages: (1) screening of titles and abstracts and (2) inclusion assessment for full-text articles.

#### 2.4.1 Stage 1: Title and abstract screening

During the initial phase, studies were retained if they met all of the following criteria:

- **Peer-reviewed source:** Published in a peer-reviewed journal or conference proceeding between January 2020 and June 2025.
- **Language:** Written in English, Portuguese, or Spanish.
- **Scope of contribution:** Explicitly describe, evaluate, or propose a software architecture for HIS or a digital-health-related software solution.
- **Originality:** Not a review, book chapter, or secondary source that did not introduce a new architectural approach.
- **Clarity:** Contained sufficient methodological or conceptual detail to understand the proposed architectural contribution (studies that were too vague or purely conceptual without an architecture description were excluded).

#### 2.4.2 Stage 2: Full-text inclusion assessment

Articles that passed the first stage were retrieved in full text and assessed against the following inclusion criteria:

- **Domain specificity:** The study explicitly addressed both healthcare and software architecture domains.
- **Information sufficiency:** The study provided adequate methodological, contextual, or design information to answer the research questions of this review.
- **Relevance of focus:** The study was not centered on a highly specific or niche topic outside the scope of the health information system architecture (e.g., isolated algorithmic or device-level implementations).

Studies that failed to meet one or more of these conditions were excluded, with reasons for exclusion depicted in the PRISMA flow diagram.

### 2.5 Data extraction

Data extraction focused on:

- Publication year
- Country of first author
- Type of creators:
  ○ Academy
  ○ Enterprise
  ○ Government
  ○ Collaborations
- Main application target (EHR, interoperability, IoT integration, etc.)
- Predominant architectural pattern
- Inclusion of digital ecosystem concepts

Data were coded using predefined categories based on similarities identified during preliminary screening. Extraction was carried out by two reviewers using a shared database to ensure consistency. Any disagreements were resolved through discussion.

### 2.6 Risk of bias assessment

To ensure methodological transparency and evaluate the credibility of the included evidence, a risk of bias assessment was conducted for all studies that met the full-text inclusion criteria.

Given the qualitative and conceptual nature of the research designs typically found in software architecture studies, the Joanna Briggs Institute (JBI) Critical Appraisal Checklist for Qualitative Research was selected as the most suitable instrument. This tool enables the appraisal of congruence between study objectives, methodological design, and reported findings.

Each article was independently assessed by two reviewers to examine the following key dimensions:

- Alignment between research objectives and study design
- Clarity of the context and representation of the phenomenon of interest
- Appropriateness of data collection and analysis methods
- Transparency in interpretation and logical support for conclusions
- Identification of potential researcher influence or interpretive bias

Discrepancies between reviewers were discussed until consensus was achieved. The individual item ratings were synthesized into a qualitative summary table (provided in the Supplementary Material), categorizing studies as having low, moderate, or high risk of bias.

The assessment informed the interpretation of findings in the Discussion section, particularly regarding the credibility, transferability, and dependability of architectural evidence across different digital health contexts.

### 2.7 Analysis plan

The analysis consisted of two strategies described in the following subsections and combined descriptive quantitative synthesis with interpretive narrative analysis to capture both the structural patterns and contextual nuances of health information system architectures.

#### 2.7.1 Quantitative analysis

Descriptive analyses were used to summarize the main characteristics of the study. Data were extracted from the review database using SQL queries, resulting in structured dataframes for statistical summaries and advanced analyses using R (v4.3).

Frequency distributions and visualizations (bar charts, pie charts, and Sankey diagrams) were generated to illustrate the relationships among the following:

- Architectural patterns;
- Application targets (e.g., EHR, interoperability, IoT, and AI-assisted health); and
- Ecosystem-level dimensions such as modularity and governance, among others.

This quantitative layer established the structural overview of the field and guided the thematic coding process for the narrative synthesis.

#### 2.7.2 Narrative synthesis

To deepen understanding beyond descriptive counts, a narrative synthesis was undertaken to interpret methodological, conceptual, and contextual dimensions reported. This process adhered to the methodological framework proposed by reference (5) for systematic narrative reviews and consisted of the following phases:

1. **Data familiarization and thematic coding:** Each full-text study was reviewed in detail for identifying the following core analytical categories: (1) pattern type, (2) interoperability mechanisms, (3) data governance, and (4) ecosystem principles.
2. **Within and cross-study exploration:** Convergence and divergence patterns were examined to identify recurring rationales behind architectural choices and contextual variations across healthcare domains and geographies, if present.
3. **Conceptual mapping and integration:** A conceptual matrix was constructed linking architecture types to implementation contexts, enabling comparison of technical strategies (e.g., FHIR adoption, blockchain frameworks) and ecosystem-level attributes (e.g., openness, sustainability, and intersectoral coordination).
4. **Triangulation and validation:** Quantitative trends from descriptive analyses were triangulated with qualitative themes to enhance credibility and dependability.

#### 2.7.3 Outcome integration

The integrated synthesis enabled the identification of dominant architectural paradigms, emerging hybrid models, and systemic gaps (e.g., AI integration, sustainability, and cross-border interoperability). These insights directly informed the interpretation and implications discussed in the Results section, ensuring that both quantitative and qualitative pieces of evidence were cohesively aligned to the research questions.

## 3 Results

### 3.1 Study selection

The search across the four databases retrieved 304 records. After removing 36 duplicates, 268 unique records were screened by title and abstract. Following a full-text assessment of 214 articles, 125 were excluded for the following reasons:

- Reviews of book chapters without new proposals (*n* = 20)
- Non-healthcare- or non-architecture-specific (*n* = 36)
- Too vague or too specific, limiting generalization (*n* = 69)

**Figure 1:**
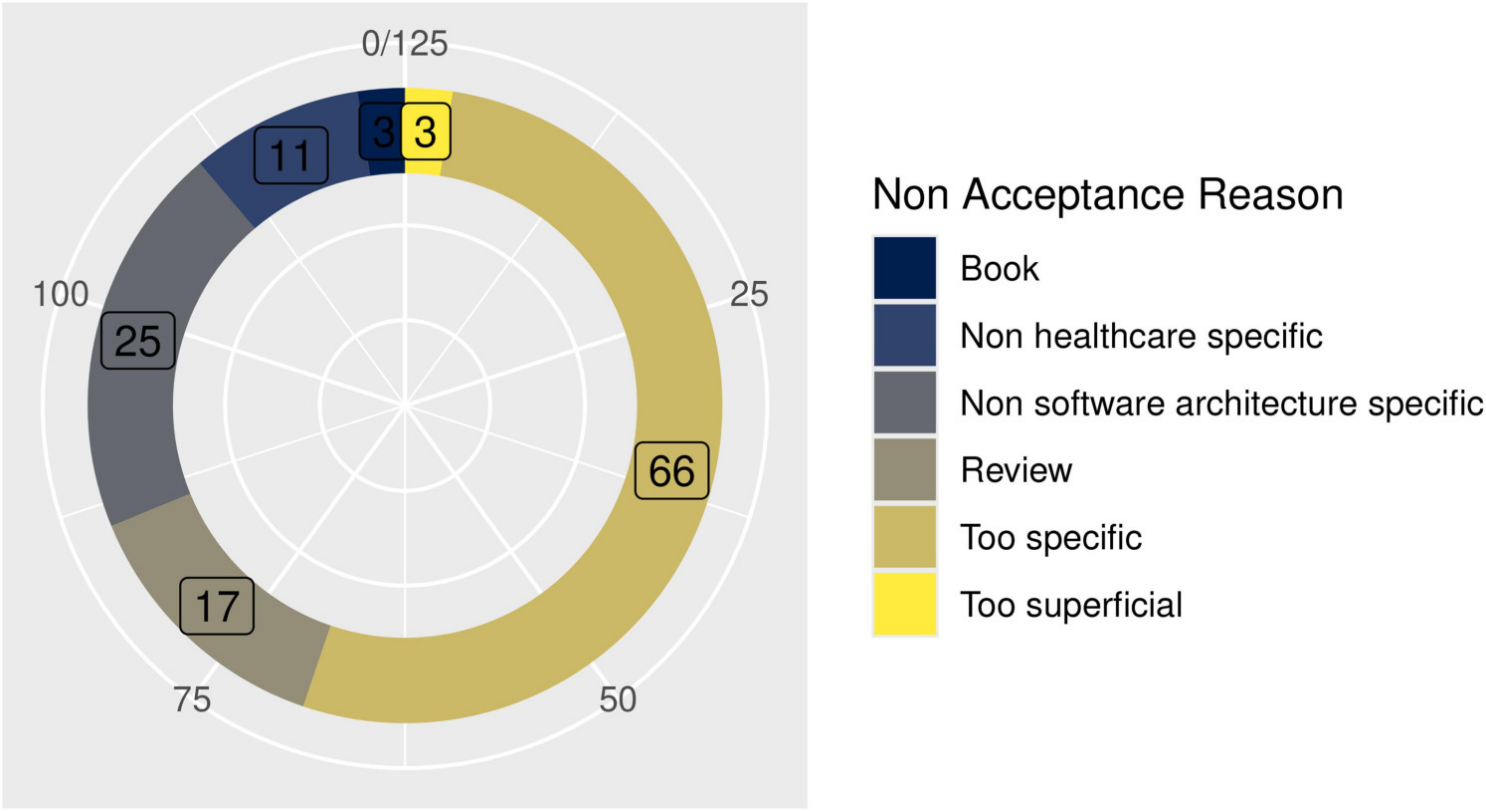
Reason for non-acceptance.

Articles or conference papers not accepted by the evaluators are listed in Table 2, categorizing each of them in its own category. Furthermore, Figure 1 also shows the distribution of reasons for non-acceptance.

**Table 2:** Discarded records and reason for non-acceptance.

Reason	*n*	%	Resources
Book chapter	3	2.4	(6–8)
Review	17	13.6	(9–24)
Non-healthcare-specific	11	8.8	(25–35)
Non-software architecture-specific	25	20	(36–60)
Too vague or too specific	69	55.2	(61–129)

**Figure 2:**
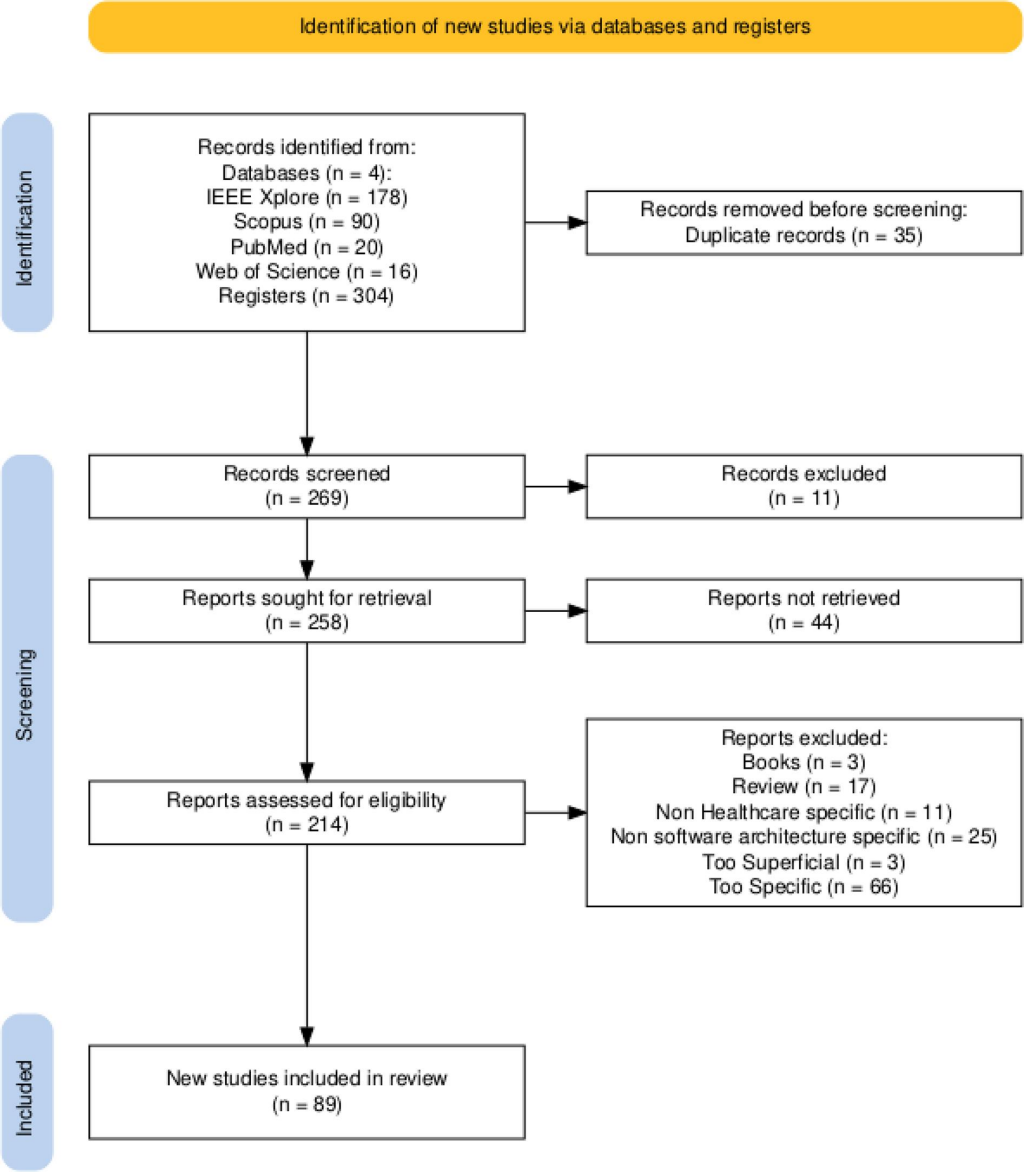
PRISMA flow diagram of the search, screening, and selection process.

The whole process is depicted in the flow diagram in Figure 2.

Ultimately, 89 studies were included for complete data extraction.

### 3.2 Characteristics of included studies

**Figure 3:**
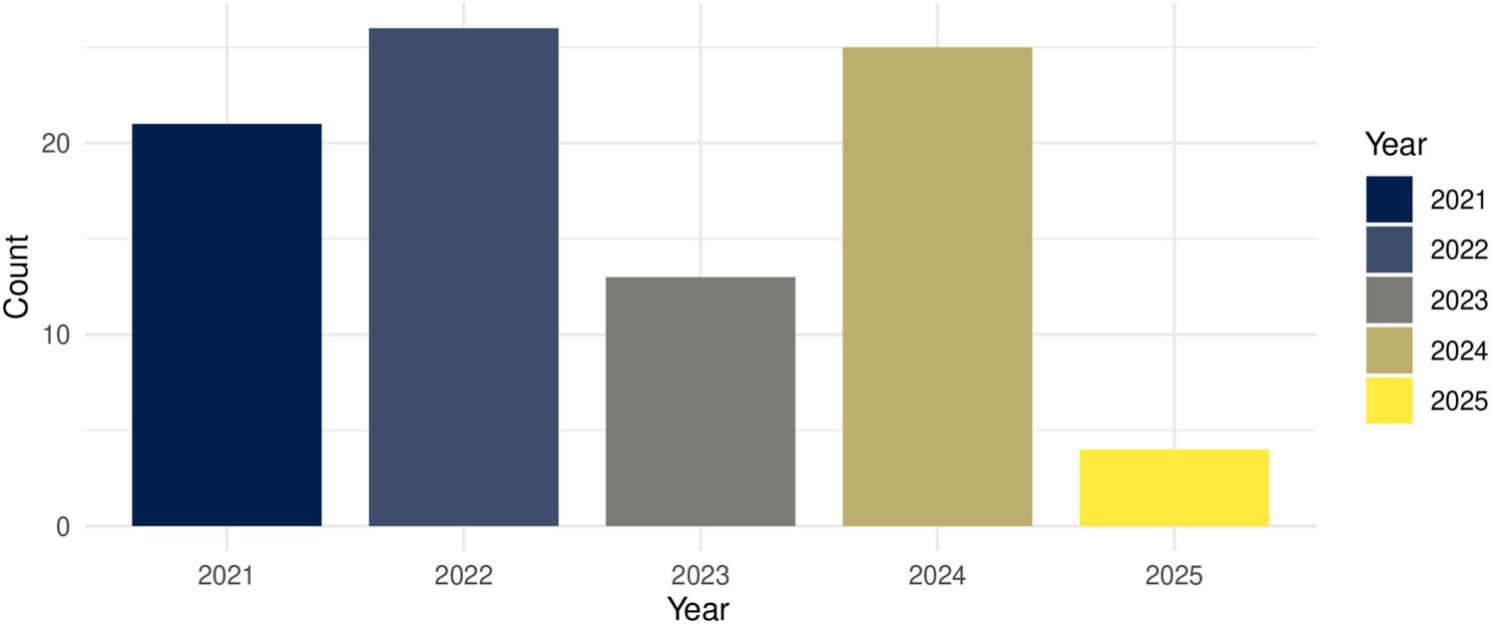
Number of published articles found per year.

The included studies were published between 2020 and 2025, with the highest number in 2020 (*n* = 26), followed by 2024 (*n* = 25) and 2021 (*n* = 21) (Figure 3).

**Figure 4:**
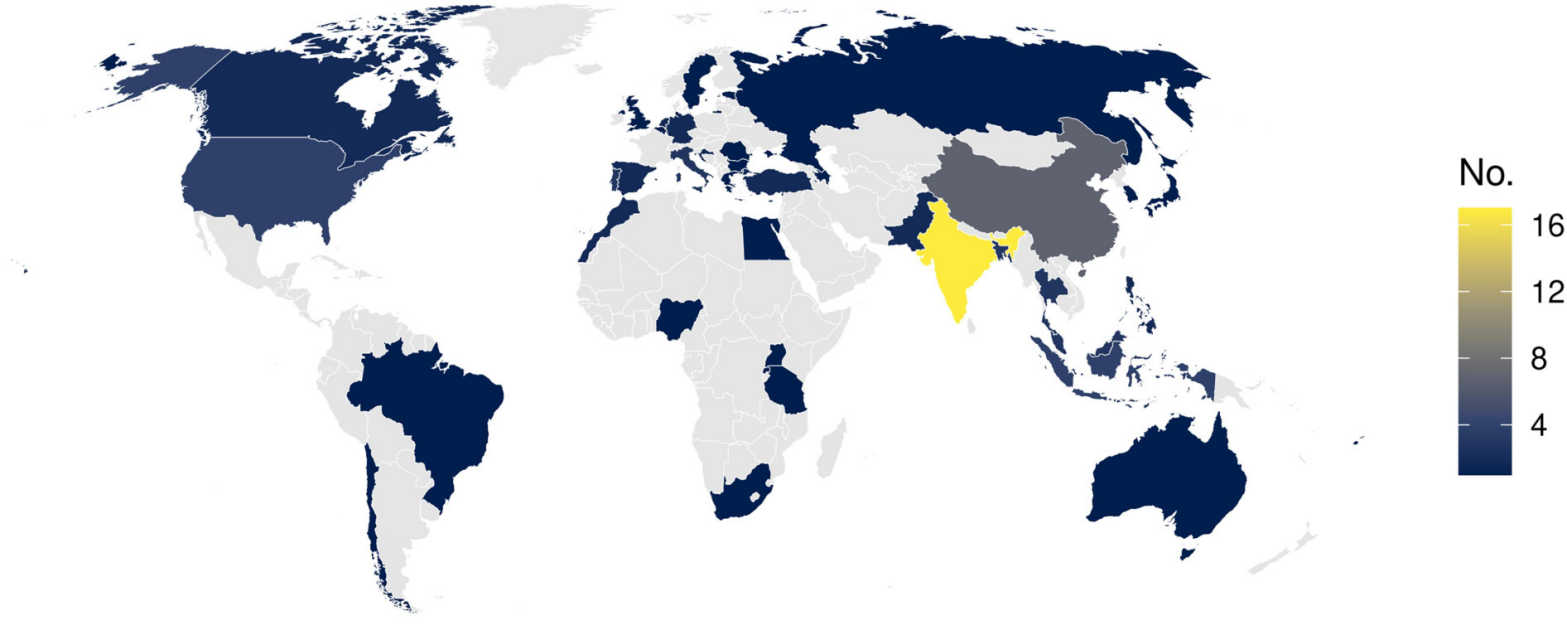
Publications by country of the main author. Map created using the rnaturalearth: World Map Data from Natural Earth package by Philippe Massicotte, Andy South and Koen Hufkens, licensed under MIT License.

Geographically, most of the contributions originated in Asia and Europe, with notable representation from India, China, and the member states of the European Union (Figure 4).

### 3.3 Main targets

**Figure 5:**
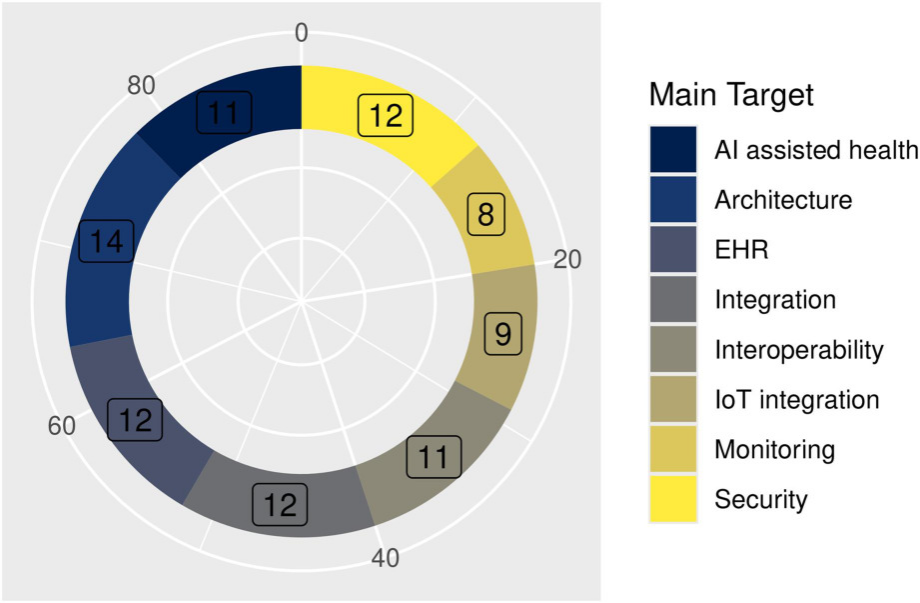
Distribution of the main target for reported solutions.

Analysis of application targets revealed eight clusters (Table 3; Figure 5). Table 3 presents the number of articles included in each group and their corresponding percentage weights.

**Table 3:** Main target cluster grouping included resources.

Main target	*n*	%	Resources
AI-assisted health	11	12.4	(130–140)
Architecture	14	15.7	(141–154)
EHR	12	13.4	(15, 155–165)
Integration	12	13.4	(166–177)
Interoperability	11	12.3	(178–188)
IoT integration	9	10.1	(189–197)
Monitoring	8	8.9	(198–204)
Security	12	13.4	(205–216)

This distribution reflects a field strongly oriented to interoperability and integration, while security and AI remain critical but unevenly addressed.

### 3.4 Predominant architecture patterns

The classification of architectural patterns based on the taxonomy proposed by O’Reilly (217) and the extended categories shows a clear dominance of the following:

- Service-oriented patterns, primarily microservices (*n* = 39; 43.8%); and
- Distributed or decentralized ledger-oriented architectures, mainly blockchain-based (*n* = 30; 33.7%).

**Figure 6:**
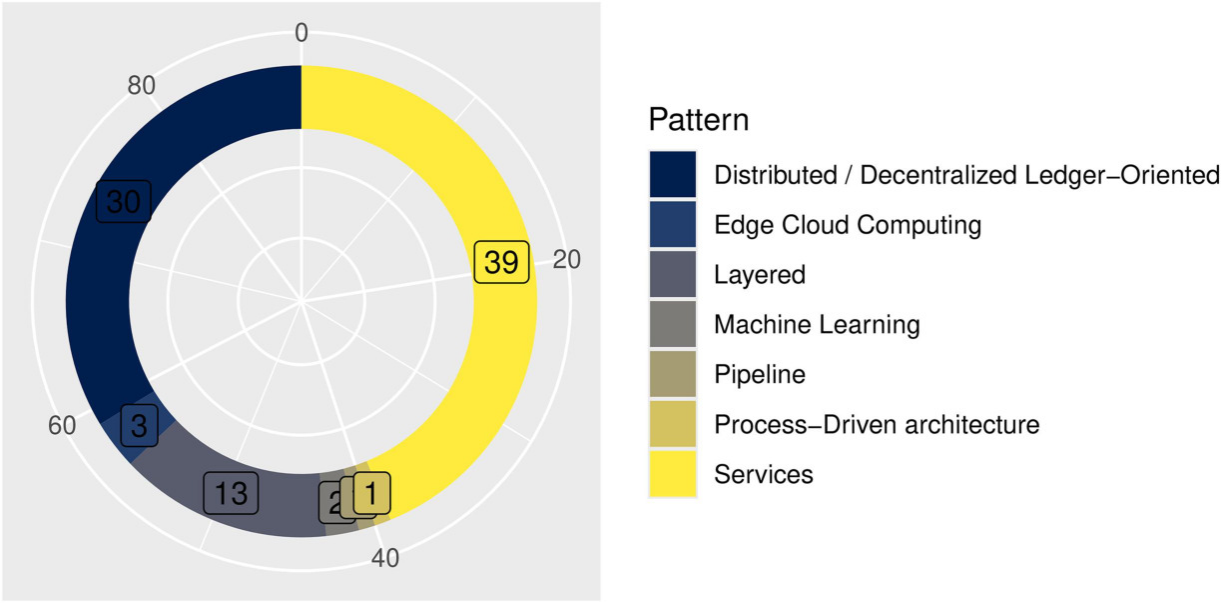
Architectural patterns of the reported solutions.

Figure 6 illustrates the distribution across architectural patterns.

Table 4 lists the number of articles included.

**Table 4:** Predominant architectural patterns reported in included resources and their main contrasting attributes.

Pattern	*n*	%	Primary focus	Technologies	Application contexts
Services	39	43.8	Modularity, interoperability, and scalability through decomposition of HIS into reusable components	HL7 FHIR, RESTful APIs, Kubernetes, Docker	Clinical data exchange, EHR interoperability, patient record management
Distributed or decentralized ledger-oriented	30	33.7	Data integrity, transparency, and trust among distributed entities	Blockchain (Ethereum, Hyperledger), smart contracts, consensus algorithms	Cross-institutional data sharing, patient consent management, audit trails
Layered	13	14.6	Separation of concerns, maintainability, and hierarchical organization of system logic	MVC frameworks, SOA, openEHR	Traditional HIS design, hospital information workflows, national EHR infrastructures
Edge-cloud computing	3	3.4	Low-latency processing and elastic scalability via distributed computation	IoT protocols (MQTT), container orchestration, edge gateways	Remote monitoring, telemedicine, IoT-enabled health analytics
Machine learning	2	2.2	Adaptive intelligence and predictive analytics within HIS	TensorFlow, PyTorch, FHIR resources for AI inputs	Clinical decision support, diagnostic prediction, image analysis
Process-driven architecture	1	1.1	Workflow automation and traceability through process orchestration	BPMN, Camunda, BPEL	Care pathways, administrative process optimization
Pipeline	1	1.1	Sequential data transformation for reproducible analytics and AI workflows	ETL frameworks, Apache Airflow, data lakes	Big data analytics, AI model training and evaluation

### 3.5 Digital health ecosystem concept

More than half of the included studies (*n* = 48; 53.9%) explicitly referenced ecosystem principles, such as openness, modularity, and interoperability, frequently framed within SECO and SoS frameworks (Table 5). These works emphasize decentralized collaboration among heterogeneous systems, adaptive governance, and the evolution of health information infrastructures through federated and standards-based integration models.

**Table 5:** Distribution of resources according to their conception of health ecosystem.

Ecosystem conception	*n*	%	Resources
Present	48	53.9	(15, 84, 136, 138, 142, 144–146, 148–156, 161, 163, 168–172, 174, 178, 179, 181–183, 186, 187, 190, 194, 195, 197, 200, 201, 205–207, 209, 210, 212–216)
Lacking	41	46.1	(86, 130–135, 137, 139–141, 143, 147, 157–160, 162, 164–167, 173, 175–177, 180, 184, 188, 189, 191–193, 196, 198, 199, 202–204, 208, 218)

Representative examples include blockchain-enhanced ecosystems for secure data sharing (156, 170), multistakeholder frameworks supporting pandemic response and population surveillance (138, 142), and architectures aligning standards with SECO principles (183, 197, 205).

In contrast, 41 (46.1%) focused primarily on technical implementation or infrastructure optimization (service orchestration improvement, data pipeline implementation, and diagnostic algorithms, among others) without situating their contributions within an ecosystemic or multiactor context. Although many demonstrated architectural innovation (130, 131, 204), they often lacked consideration of cross-organizational governance, sustainability, or policy interoperability. This imbalance highlights a persistent gap between technical performance and ecosystem maturity, suggesting that ecosystem awareness remains an evolving frontier in HIS research.

**Figure 7:**
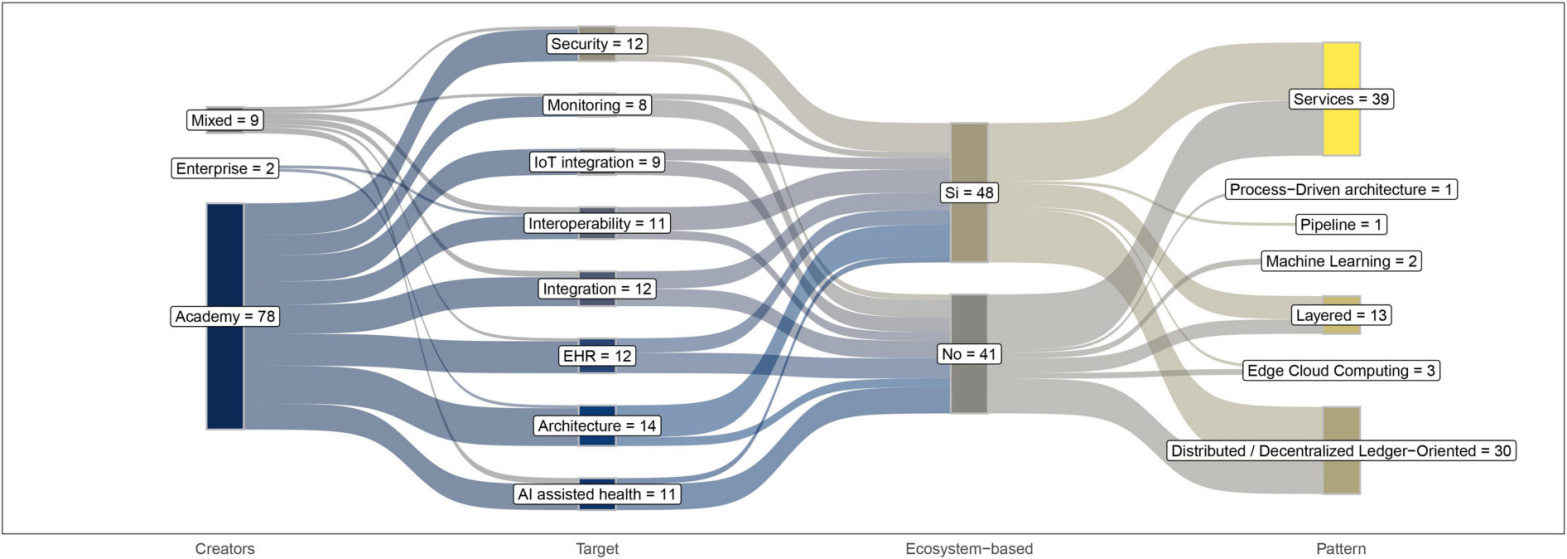
Sankey diagram illustrating the flow of outcomes and their distribution across relevance categorization.

The relationships among targets, patterns, and ecosystem inclusion are shown in Figure 7.

## 4 Discussion

### 4.1 Principal findings

This systematic review identified 89 studies addressing software architectures in HIS. The analysis revealed that microservice-based architectures dominate current implementations, followed by distributed or decentralized ledger-oriented solutions, mainly blockchain. In addition, more than half of the studies referenced ecosystem-level concepts such as interoperability and modularity, although a significant portion remained focused on isolated technical solutions. Emerging approaches, including edge-cloud integration and machine learning-based architectures, have been appeared in niche use cases.

### 4.2 Interpretation and implications

The dominance of microservice-based architectures reflects a strong orientation toward flexibility, scalability, and modularity, attributes critical for dynamic healthcare environments. These architectures enable incremental innovation and align well with interoperability standards such as HL7 FHIR, which act as a stabilizing interface across heterogeneous systems (159, 197). Evidence suggests that microservices not only support modularity but also reduce vendor lock-in and foster resilience through uncoupled components, allowing for the seamless integration of emerging technologies (210).

Equally significant is the growing application of distributed or decentralized ledger-oriented architectures, primarily blockchain, as a response to escalating concerns around data security, confidentiality, and traceability (205, 219). These approaches provide immutable transaction logs and enable granular access control, enhancing trust among stakeholders. However, practical constraints, such as energy consumption, latency, and scalability limitations, hinder their large-scale adoption in healthcare settings (69, 70). Consequently, hybrid strategies that integrate blockchain with service-oriented patterns or edge computing frameworks are likely to emerge as viable alternatives.

Beyond patterns, our analysis of application targets reveals an ecosystem under pressure to respond to multifaceted demands:

- Artificial intelligence-assisted health solutions are being explored for advanced diagnostic support and decision-making; however, data heterogeneity and governance gaps remain critical barriers (130, 131). The literature emphasizes the urgent need for reference architectures tailored for AI integration into interoperable HIS platforms (134).
- Data integration architectures seek to consolidate clinical, genomic, and administrative sources, yet progress is constrained by semantic inconsistencies and a lack of standardized ontologies (64, 65).
- IoT- and IoMT-enabled monitoring solutions promise real-time analytics for preventive care but introduce interoperability, processing load, and cybersecurity challenges that require standardized protocols and lightweight security layers (77, 220).
- Persistent attention to security frameworks illustrates a sector grappling with cyber vulnerabilities. While blockchain offers partial solutions, the evidence suggests that multilayered strategies incorporating encryption, multifactor authentication, and governance policies are more effective (75, 82).

The implications of these findings are clear: architectural design choices must go beyond technical optimization to incorporate governance, ethics, and sustainability, positioning HIS as enablers of resilient and adaptive digital health ecosystems.

### 4.3 Contrast with existing literature

Consistent with experiences reported in JAMIA, where systematic reviews have historically guided international adoption of HIS standards, our findings confirm the importance of architectural synthesis for evidence-based adoption.

Previous systematic reviews have consistently underscored the value of service-oriented architectures for fostering interoperability and reducing operational complexity (81, 197). Our findings corroborate these observations but extend them by identifying a trend toward hybridization, where microservices are complemented by event-driven mechanisms to support real-time responsiveness and edge computing layers to minimize latency in mission-critical applications (62, 67). These hybrid models align with the emerging discourse on decentralized digital ecosystems, where adaptability and scalability are paramount.

The integration of AI-driven services within HIS, although recognized in earlier studies (132, 133), remains an emergent capability rather than a standard feature. Our synthesis emphasizes that successful adoption hinges on addressing data governance frameworks and establishing normative clarity for clinical-grade AI deployments.

Similarly, while blockchain-based approaches have been celebrated for their auditability and security guarantees, earlier reviews rarely confront the operational constraints our analysis highlights, such as computational overhead and regulatory inertia (210, 219). These limitations reinforce the argument that fully decentralized models may be impractical for healthcare unless combined with other architectural patterns, such as layered or service-oriented architectures.

Finally, our review supports and expands on the literature’s consensus that interoperability challenges are less technical than organizational. Frameworks like HL7 FHIR and OpenEHR continue to dominate as enablers of semantic and structural consistency (74, 81); however, adoption remains uneven across different geographies. This suggests that future architectural strategies should embed policy-aware interoperability mechanisms alongside technical layers to bridge global disparities.

### 4.4 Strengths and limitations

This review systematically synthesized evidence from major academic databases, applying PRISMA guidelines to ensure rigor. However, it is subject to certain limitations:

- Publication bias: The predominance of academic contributions may underrepresent non-academic innovations (204, 221–223).
- Language restriction: Only studies in English, Spanish, or Portuguese were included.
- Scope of analysis: The review focused on architectural descriptions, without assessing the effectiveness of implementations in clinical settings.

### 4.5 Future research directions

Given the stated findings, future investigation should focus on developing reference architectures explicitly incorporating ecosystem governance, data ethics, and cross-border interoperability. Furthermore, it is important to refine blockchain alternatives, integrating them into hybrid models to overcome current scalability challenges.

Research should also focus on developing data management policies and governance frameworks to enable the safe integration of artificial intelligence applications into health information systems in production environments.

## 5 Conclusions

This review synthesized evidence from 89 studies and confirmed that microservices (159, 197) and distributed ledger approaches (205, 210, 219) dominate the architectural landscape of health information systems. Both patterns align with the need for scalability, interoperability, and trust, particularly when combined with standards such as HL7 FHIR and OpenEHR (74, 81). These findings confirm a strong trend toward hybrid architectures, where microservices and event-driven mechanisms integrate with blockchain, layered, or edge-computing solutions to deliver flexible and resilient infrastructure.

At the same time, our analysis revealed significant gaps in cross-border care, sustainability, and the integration of artificial intelligence. For cross-border healthcare, initiatives such as epSOS and X-Road illustrate partial progress in secure data exchange but still struggle with semantic interoperability and governance heterogeneity across national contexts (224). Despite FHIR’s convergence potential, uneven implementation and limited semantic alignment hinder continuity of care beyond local environments. Furthermore, although modular and cloud-native architectures enhance performance and scalability, long-term sustainability is rarely evaluated, particularly in low- and middle-income contexts where infrastructure, funding, and maintenance capacities are uneven (81). Studies highlight how microservices can support modular encapsulation and even integrate IoT-based environmental sensing into health big data platforms (225); however, these efforts remain isolated. A significant issue is the lack of governance models for managing the life cycle of the microservices and ensuring energy-efficient operation (226).

Regarding AI integration, despite significant advances in diagnostic and decision-support applications (130, 131), implementations are still fragmented, largely decoupled from HIS workflows, and constrained by data heterogeneity and the absence of reference models (132–134). Initiatives such as the World Health Organization’s Artificial Intelligence for Health framework underscore the need for transparent governance and continuous evaluation to ensure trustworthy adoption of AI within SoS and SECO paradigms (227, 228).

The implications for practice are clear: healthcare organizations should pursue multipattern strategies in which microservices support modularity, blockchain enhances security and traceability, and edge-cloud integration ensures real-time responsiveness (62, 67). Yet, architecture alone is insufficient. Adoption depends on synchronized standards, semantics, usability, and data governance mechanisms that foster scalability and trust across institutions (229). Event-driven and lazy-coupling strategies emerge as enablers of resilience and responsiveness, particularly when aligned with interoperable service and edge architectures (230, 231).

The experience of JAMIA demonstrates how systematic reviews of HIS architectures can consolidate best practices and inform policy translation. By integrating fragmented evidence, this review reinforces the call for reference architectures that explicitly embed governance, ethics, and sustainability principles. The combination of FHIR contracts with one or multiple architectural patterns has proven to stabilize interfaces and simplify the incorporation of new applications (232–234).

Finally, the future of HIS will not depend on a single dominant paradigm but on the intentional orchestration of diverse architectural patterns within open, sustainable, and trustworthy ecosystems.

## Data Availability

The original contributions presented in the study are included in the article/Supplementary Material; further inquiries can be directed to the corresponding author.

## References

[1] Schlieter H Benedict M Gand K Burwitz M. Towards adaptive pathways: reference architecture for personalized dynamic pathways. In: *2017 IEEE 19th Conference on Business Informatics (CBI)*. (2017). Vol. 1. p. 359–68.

[2] Syed R Eden R Makasi T Chukwudi I Mamudu A Kamalpour M Digital health data quality issues: systematic review. J Med Internet Res. (2023) 25:e42615. 10.2196/42615 37000497 PMC10131725

[3] Mohsin Khan M Shah N Shaikh N Thabet A. Towards secure and trusted AI in healthcare: a systematic review of emerging innovations and ethical challenges. Int J Med Inform. (2025) 195:105780. 10.1016/j.ijmedinf.2024.105780 39753062

[4] Page MJ McKenzie JE Bossuyt PM Boutron I Hoffmann TC Mulrow CD The PRISMA 2020 statement: an updated guideline for reporting systematic reviews. BMJ. (2021) 372:n71. 10.1136/bmj.n71 33782057 PMC8005924

[5] Popay J Roberts H Sowden A Petticrew M Arai L Rodgers M Guidance on the Conduct of Narrative Synthesis in Systematic Reviews: A Product from the ESRC Methods Programme. Lancaster: Institute of Health Research (2006). 10.13140/2.1.1018.4643

[6] Abbasi S. White Paper—Overview and Insight: Performance of Digital Health Systems during the COVID-19 Pandemic. New York: IEEE (2023).

[7] Pereira JD Brito MA Machado RJ. Development of an Interoperability Platform for Information Systems in the e-Health Domain, for the Portuguese Health System. Lancaster, NY: Curran Associates, Inc. (2023).

[8] Merzweiler A Staubert S Strübing A Tonmbiak A Kaulke K Drepper J The process of modeling information system architectures with IHE. Stud Health Technol Inform. (2021) 278:163–70. 10.3233/SHTI210065 34042890

[9] Rohith M Sunil A Mohana. Comparative analysis of edge computing and edge devices: key technology in IoT and computer vision applications. In: *2021 6th International Conference on Recent Trends on Electronics, Information, Communication and Technology, RTEICT 2021*. Institute of Electrical and Electronics Engineers Inc. (2021). p. 722–7.

[10] Haddad T Kumarapeli P de Lusignan S. Barriers and opportunities in developing health information system (HIS) architectures to support universal health coverage in resource-limited settings. Stud Health Technol Inform. (2025) 327:363–7. 10.3233/SHTI250345. 40380456

[11] Haux R. Health information systems: past, present, future-revisited. In: *Studies in Health Technology and Informatics*. IOS Press BV (2022). Vol. 300. p. 108–34. 10.3233/SHTI220945 36300406

[12] Condry MW. Using requirements for health data organization and management. IEEE Eng Manage Rev. (2021) 49:109–18. 10.1109/EMR.2021.3100418

[13] Ali KA Alyounis S. Cybersecurity in healthcare industry. In: *2021 International Conference on Information Technology, ICIT 2021—Proceedings*. Institute of Electrical and Electronics Engineers Inc. (2021). p. 695–701.

[14] Chen F Tang Y Wang C Huang J Huang C Xie D Medical cyber-physical systems: a solution to smart health and the state of the art. IEEE Trans Comput Soc Syst. (2022) 9:1359–86. 10.1109/TCSS.2021.3122807

[15] Arsheen S Ahmad K. Blockchain-enabled immunization system: a novel idea to leverage reliability and traceability. In: *2022 3rd International Conference for Emerging Technology, INCET 2022*. Institute of Electrical and Electronics Engineers Inc. (2022).

[16] Gulzar M Ahmed M. In: *2023 10th International Conference on Computing for Sustainable Global Development*. IEEE (2023).

[17] Gupta A Gupta U Kumar A Bhushan B. Analysing security threats and elevating healthcare privacy for a resilient future. In: *International Conference on Artificial Intelligence for Innovations in Healthcare Industries, ICAIIHI 2023*. Institute of Electrical and Electronics Engineers Inc. (2023).

[18] Harahap NC Handayani PW Hidayanto AN. The challenges in integrated personal health record adoption in Indonesia: a qualitative analysis of regulatory perspectives. In: *Proceedings—3rd International Conference on Informatics, Multimedia, Cyber, and Information System, ICIMCIS 2021*. Institute of Electrical and Electronics Engineers Inc. (2021). p. 169–74.

[19] Talpur MSH Abro AA Ebrahim M Kandhro IA Manickam S Laghari SUA Illuminating healthcare management: a comprehensive review of IoT-enabled chronic disease monitoring. IEEE Access. (2024) 12:48189–209. 10.1109/ACCESS.2024.3382011

[20] Khatri S Alzahrani FA Ansari MTJ Agrawal A Kumar R Khan RA. A systematic analysis on blockchain integration with healthcare domain: scope and challenges. IEEE Access. (2021) 9:84666–87. 10.1109/ACCESS.2021.3087608

[21] Haleem A Javaid M Singh RP Suman R Rab S. Blockchain technology applications in healthcare: an overview. Int J Intell Netw. (2021) 2:130–9. 10.1016/j.ijin.2021.09.005

[22] Quintero V Chevel C Sanmartin-Mendoza P. Analysis on the interoperability of health information systems. In: *2024 IEEE Technology and Engineering Management Society, TEMSCON LATAM 2024*. Institute of Electrical and Electronics Engineers Inc. (2024).

[23] Sitompul T Hendric Spits Warnars HL Meyliana Hidayanto AN Prabowo H. The country’s implementation and adoption of standardized health terminologies to promote interoperability: a systematic literature review. In: *Proceedings of 2023 International Conference on Information Management and Technology, ICIMTech 2023*. Institute of Electrical and Electronics Engineers Inc. (2023). p. 766–71.

[24] Verma R Bhatt R. Security in big data health care system. In: *Proceedings of the IEEE International Conference Image Information Processing*. Institute of Electrical and Electronics Engineers Inc. (2021). p. 551–5.

[25] Adetiba E Nnaji U Wejin JS Ojesanmi F Akanle MB Ajayi PO FedMonitor: an implementation architecture for monitoring of resources and events’notifications on federated cloud computing infrastructures. In: *2024 International Conference on Emerging Trends in Networks and Computer Communications (ETNCC)*. IEEE (2024). p. 1–6.

[26] Kogias DG Patrikakis CZ. Beyond bitcoin: exploring the expanding horizons of blockchain innovation. IT Prof. (2024) 26:4–8. 10.1109/MITP.2024.3404228

[27] Kargili OB Okan Arik A Bekler M Uygar Kose O Aktas MS. A novel distributed software architecture for managing customer behavior data: a case study in banking sector. In: *2021 21st International Conference on Computational Science and Its Applications (ICCSA)*. IEEE (2021). p. 211–7.

[28] Bertia A Xavier SB Kathrine GJW Palmer GM. A study about detecting ransomware by using different algorithms. In: *2022 International Conference on Applied Artificial Intelligence and Computing (ICAAIC)*. IEEE (2022). p. 1293–300.

[29] Narang NK. Mentor’s musings on the role of disruptive technologies and innovation in making healthcare systems more sustainable. IEEE Internet Things Mag. (2021) 4:80–9. 10.1109/MIOT.2021.9548847

[30] Abbas J Zhang C Luo B. EnsCL-CatBoost: a strategic framework for software requirements classification. IEEE Access. (2024) 12:127614–28. 10.1109/ACCESS.2024.3452011

[31] Mwakabira IS Byson LF Manda TD Phiri YD Simwela A. Design gaps in configurable systems: adaptability of DHIS2 to other domains. In: *2023 IST-Africa Conference (IST-Africa)*. IEEE (2023). p. 1–8.

[32] Tang X Zheng X Wang X Huang J. A privacy-preserving surface defect detection scheme for strip steel. In: *2023 38th Youth Academic Annual Conference of Chinese Association of Automation (YAC)*. IEEE (2023). p. 486–93.

[33] Bozkurt Y Rossmann A Konanahalli A Pervez Z. Toward urban data governance: status-quo, challenges, and success factors. IEEE Access. (2023) 11:85656–77. 10.1109/ACCESS.2023.3302835

[34] Habibah IA Hasibuan LS Giri EP Adrianto HA. SiTernak mobile application development to report livestock health data. In: *International Conference on Computer, Control, Informatics and its Applications, IC3INA*. Institute of Electrical and Electronics Engineers Inc. (2024). Vol. 2024. p. 18–23.

[35] Abubaker I Owda M Owda AY. Multi-channel fusion model for data logs analysis and anomaly detection in data centers. In: *2024 4th International Conference of Science and Information Technology in Smart Administration (ICSINTESA)*. IEEE (2024). p. 546–51.

[36] Sonia Dewi PP Lubis M Abdurrahman L. Aligning healthcare strategies through digital information and technology: challenges and solution. In: *2024 12th International Conference on Cyber and IT Service Management, CITSM 2024*. Institute of Electrical and Electronics Engineers Inc. (2024).

[37] Jyothirmai D Vineela B Pitchai R Dinesh B Indhu M Avinash A. Data leakage detection using secret key exchange. In: *2023 2nd International Conference on Edge Computing and Applications (ICECAA)*. IEEE (2023). p. 218–22.

[38] Abramowitz S Stevens LA Kyomba G Mayaka S Grépin KA. Data flows during public health emergencies in LMICs: a people-centered mapping of data flows during the 2018 ebola epidemic in Equateur, DRC. Soc Sci Med. (2023) 318:115116. 10.1016/j.socscimed.2022.115116 36610244

[39] Harrison S Maple C Epiphaniou G Arvanitis TN. Improving quality in digital health intervention safety (extended abstract). In: *IET Conference Proceedings*. Institution of Engineering and Technology (2023). Vol. 2023. p. 164–9.

[40] Vazirian S Ho T Weideman RA Salinas MR Hurd PW Stuve O. Utilization of a neurology specialty service by primary care providers for headache management at a tertiary care hospital. J Cent Nerv Syst Dis. (2022) 14:11795735221113102. 10.1177/11795735221113102 35860714 PMC9290155

[41] Gorvunova V Kukhtevich I Goryunova T. Digitalization and integration cloud solutions for healthcare information systems. In: *Proceedings—2022 4th International Conference on Control Systems, Mathematical Modeling, Automation and Energy Efficiency, SUMMA 2022*. Institute of Electrical and Electronics Engineers Inc. (2022). p. 608–11.

[42] Gerasimov A Trofimov E Kopanitsa G Metsker O. Modelling investment programs with machine learning and data mining: descriptive and predictive models of healthcare state programs in Russia. In: *2022 31st Conference of Open Innovations Association (FRUCT)*. IEEE (2022). p. 45–50.

[43] Ebardo R Fermin ML. Sociotechnical barriers to data privacy compliance in Philippine healthcare. In: *2023 IEEE 15th International Conference on Humanoid, Nanotechnology, Information Technology, Communication and Control, Environment, and Management, HNICEM 2023*. Institute of Electrical and Electronics Engineers Inc. (2023).

[44] Darwish SM Essa RM Osman MA Ismail AA. Privacy preserving data mining framework for negative association rules: an application to healthcare informatics. IEEE Access. (2022) 10:76268–80. 10.1109/ACCESS.2022.3192447

[45] Cai W Gao M Jiang Y Gu X Ning X Qian P Hierarchical domain adaptation projective dictionary pair learning model for EEG classification in IoMT systems. IEEE Trans Comput Soc Syst. (2023) 10:1559–67. 10.1109/TCSS.2022.3176656

[46] Boyle C Madevu-Matson C Rachmanto A Kunaka D Gilliss L McManus L Does investment in COVID-19 information systems strengthen national digital health architecture? Lessons learned from Burkina Faso, Indonesia, Mali and Suriname. Oxf Open Digit Health. (2024) 2:i29–39. 10.1093/oodh/oqae001 40230395 PMC11935742

[47] Kukhtevich I Goryunova V Goryunova T. Aspects of using intelligent cloud technologies in professional training of general practitioners. In: *2021 1st International Conference on Technology Enhanced Learning in Higher Education (TELE)*. IEEE (2021). p. 53–7.

[48] Rojas AJS Valencia EFP Armas-Aguirre J Molina JMM. Cybersecurity maturity model for the protection and privacy of personal health data. In: *2022 IEEE 2nd International Conference on Advanced Learning Technologies on Education & Research (ICALTER)*. IEEE (2022). p. 1–4.

[49] Hanson J Hui M Strawbridge JC Chatterjee S Goodyear K Giaconi JA High rates of eye surgery cancellation in veterans related to mental health. Mil Med. (2024) 189:e2588–93. 10.1093/milmed/usae230 38687601

[50] Shariat N Kargari M Alavi M Shariat S Valiollahi A. Neonatal mortality prediction in NICUs: a machine learning approach. In: *2024 10th International Conference on Web Research (ICWR)*. IEEE (2024). p. 231–7.

[51] Ndlovu K Mars M Scott RE. Validation of an interoperability framework for linking mhealth apps to electronic record systems in botswana: expert survey study. JMIR Formative Res. (2023) 7:e41225. 10.2196/41225 PMC10189626 37129939

[52] Palawat N Kiattisin S Mayakul T. A smart prevention management in gestational diabetes mellitus. In: *2022 IEEE Global Humanitarian Technology Conference (GHTC)*. IEEE (2022). p. 130–6.

[53] Odeh Y Tbaishat D Shamieh O Odeh M. Critical analytical insights of palliative care process modelling in a regional cancer care. In: *2022 International Arab Conference on Information Technology (ACIT)*. IEEE (2022). p. 1–9.

[54] Morris LD Nyatuka DR Ogundaini OO de La Harpe R Siva F Murugu DK. Towards a person-centred maternal and child health information system framework for sustained African health security using Kenya as an ICT4D case. In: *2023 IST-Africa Conference (IST-Africa)*. IEEE (2023). p. 1–12.

[55] Lee CS Ng CH Lee JS Ngo KX Teh SM Mohamed Ikraam AB Model-based customer-relationship management system and strategic board game: analogical training. In: *Proceedings—2021 3rd International Workshop on Artificial Intelligence and Education, WAIE 2021*. Institute of Electrical and Electronics Engineers Inc. (2021). p. 69–73.

[56] Kuo ZM Chen KF Tseng YJ. MoCab: a framework for the deployment of machine learning models across health information systems. Comput Methods Programs Biomed. (2024) 255:108336. 10.1016/j.cmpb.2024.108336 39079482

[57] Faroun H Zary N Baqer K Alkhaja F Gad K Alameddine M Identification of key factors for optimized health care services: protocol for a multiphase study of the Dubai vaccination campaign. JMIR Res Protoc. (2023) 12:e42278. 10.2196/42278 36541889 PMC10131770

[58] Zhao M Wang H Li Y Zhang C. A health management system for forest musk deer based on YOLOv5. In: *2023 26th ACIS International Winter Conference on Software Engineering, Artificial Intelligence, Networking and Parallel/Distributed Computing (SNPD-Winter)*. IEEE (2023). p. 20–4.

[59] McCarthy S O’Raghallaigh P Kelleher C Adam F. A socio-cognitive perspective of knowledge integration in digital innovation networks. J Strateg Inform Syst. (2025) 34:101871. 10.1016/j.jsis.2024.101871

[60] Şık AS Aydınoğlu AU Aydın Son Y. Assessing the readiness of Turkish health information systems for integrating genetic/genomic patient data: system architecture and available terminologies, legislative, and protection of personal data. Health Policy. (2021) 125:203–12. 10.1016/j.healthpol.2020.12.004 33342546

[61] Elsner B Winter A Lüdtke K Degen-Plöger C Jahn F. A strategic plan for a physiotherapy information system in a German medical center. Stud Health Technol Inform. (2025) 327:747–8. 10.3233/SHTI250449 40380558

[62] Holl F Clarke L Raffort T Serres E Archer L Saaristo P. The red cross red crescent health information system (RCHIS): an electronic medical records and health information management system for the red cross red crescent emergency response units. Confl Health. (2024) 18:28. 10.1186/s13031-024-00585-6 38589881 PMC11000295

[63] Bushra SN Shobana G. Paediatric sickle cell detection using deep learning—a review. In: *2021 International Conference on Artificial Intelligence and Smart Systems (ICAIS)*. IEEE (2021). p. 177–83.

[64] Okoroafor SC Oaiya AI Oviaesu D Ahmat A Osubor M Nyoni J. Conceptualizing and implementing a health workforce registry in Nigeria. Hum Resour Health. (2022) 20:8. 10.1186/s12960-022-00706-3 35033109 PMC8761262

[65] Khorrami F Ahmadi M Karami NA Alipour J Sheikhtaheri A. A framework for selection of health terminology systems: a prerequisite for interoperability of health information systems. Inform Med Unlocked. (2022) 31:100949. 10.1016/j.imu.2022.100949

[66] Xia Y Xue H Zhang D Mumtaz S Xu X Rodrigues JJPC. Dynamic pricing based near-optimal resource allocation for elastic edge offloading. IEEE Trans Mobile Comput. (2025) 24:8057–70. 10.1109/TMC.2025.3553188

[67] Xing J Zhangy X Jiangz Z Zhangz R Zhaz C Yinz H. Exploring investment strategies for federated learning infrastructure in medical care. In: *2021 IEEE 24th International Conference on Computational Science and Engineering (CSE)*. IEEE (2021). p. 177–84.

[68] Habib PI Murdha AS Agus H Suhardi. Architecture migration from monolithic to microservices: developing readiness criteria. IEEE Access. (2024) 12:194630–45. 10.1109/ACCESS.2024.3504848

[69] Jiang M Nakamoto I Zhuang W Zhang W Guo Y Ma L. Flexible investment strategies for cloud-native architecture of public health information systems. Wirel Commun Mobile Comput. (2021) 2021:1–11. 10.1155/2021/6676428

[70] Xue H Xia Y Xiong NN Zhang D Pei S. DDPS: dynamic differential pricing-based edge offloading system with energy harvesting devices. IEEE Trans Netw Sci Eng. (2025) 12:2549–65. 10.1109/TNSE.2025.3550251

[71] Sharma S Jain S. OntoXAI: a semantic web rule language approach for explainable artificial intelligence. Cluster Comput. (2024) 27:14951–75. 10.1007/s10586-024-04682-2

[72] Hossain MI Hasan R. Improving security practices in health information systems with STRIDE threat modeling. In: *2023 IEEE World Forum on Internet of Things: The Blue Planet: A Marriage of Sea and Space, WF-IoT 2023*. Institute of Electrical and Electronics Engineers Inc. (2023).

[73] Yao Y Zhao Z Chang X Misic J Misic VB Wang J. A novel privacy-preserving neural network computing approach for e-health information system. In: *ICC 2021—IEEE International Conference on Communications*. IEEE (2021). p. 1–6.

[74] Janith K Iddagoda R Gunawardena C Sankalpa K Abeywardena KY Yapa K. SentinelPlus: a cost-effective cyber security solution for healthcare organizations. In: *ICAC 2021—3rd International Conference on Advancements in Computing, Proceedings*. Institute of Electrical and Electronics Engineers Inc. (2021). p. 359–64.

[75] Jha S Tripathy D. Low latency consistency based protocol for fog computing systems using CoAP with machine learning. In: *2023 2nd International Conference for Innovation in Technology, INOCON 2023*. Institute of Electrical and Electronics Engineers Inc. (2023).

[76] Niu H Omitaomu OA Langston MA Olama M Ozmen O Klasky HB EHR-BERT: a BERT-based model for effective anomaly detection in electronic health records. J Biomed Inform. (2024) 150:104605. 10.1016/j.jbi.2024.104605 38331082

[77] Kanimozhi M Yamuna I Srimathi B Senthil Kumaran R. Development of health monitoring vest using velostat. In: *2021 International Conference on System, Computation, Automation and Networking, ICSCAN 2021*. Institute of Electrical and Electronics Engineers Inc. (2021).

[78] Kanjo C. Community of Practice in Practice: Successful Implementation of Integrated Community Health Information Systems. Zomba: IEEE (2022). Vol. 76.

[79] Shukla M Lin J Seneviratne O. BlockIoT-RETEL: blockchain and IoT based read-execute-transact-erase-loop environment for integrating personal health data. In: *2021 IEEE International Conference on Blockchain (Blockchain)*. IEEE (2021). p. 237–43.

[80] Nyangena J Rajgopal R Ombech EA Oloo E Luchetu H Wambugu S Maturity assessment of Kenya’s health information system interoperability readiness. BMJ Health Care Inform. (2021) 28:e100241. 10.1136/bmjhci-2020-100241 34210718 PMC8252685

[81] Bossenko I Linna K Piho G Ross P. Migration from HL7 clinical document architecture (CDA) to fast health interoperability resources (FHIR) in infectious disease information system of Estonia. In: *Proceedings of the 38th ACM/SIGAPP Symposium on Applied Computing*. New York, NY, USA: ACM (2023). p. 882–5.

[82] Kasi A Obaidat MS Rewal P Mishra D Hsiao KF. On the security of authenticated key agreement schemes for e-healthcare. In: *2022 International Conference on Communications, Computing, Cybersecurity, and Informatics (CCCI)*. IEEE (2022). p. 1–6.

[83] Yeng PK Diekuu JB Abomhara M Elhadj B Yakubu MA Oppong IN HEALER2: a framework for secure data lake towards healthcare digital transformation efforts in low and middle-income countries. In: *2023 International Conference on Emerging Trends in Networks and Computer Communications (ETNCC)*. IEEE (2023). p. 1–9.

[84] Koren A Prasad R. IoT health data in electronic health records (EHR): security and privacy issues in Era of 6G. J ICT Stand. (2022) 10:63–84. 10.13052/jicts2245-800X.1014

[85] Zaki M Sivakumar V Shrivastava S Gaurav K. Cybersecurity framework for healthcare industry using NGFW. In: *2021 Third International Conference on Intelligent Communication Technologies and Virtual Mobile Networks (ICICV)*. IEEE (2021). p. 196–200.

[86] Kumhar M Bhatia J Jadav NK Padaria AA Gupta R Tanwar S HEAL-SDN: artificial neural network-based secure data exchange framework for SDN controllers in healthcare 4.0. In: *2023 IEEE Globecom Workshops (GC Wkshps)*. IEEE (2023). p. 1832–7.

[87] Lee D Song M. MEXchange: a privacy-preserving blockchain-based framework for health information exchange using ring signature and stealth address. IEEE Access. (2021) 9:158122–39. 10.1109/ACCESS.2021.3130552

[88] Noran O Bernus P. Towards an evaluation framework for ubiquitous, self-evolving patient identification solutions in health information systems. Procedia Comput Sci. (2022) 196:550–60. 10.1016/j.procs.2021.12.048

[89] Mehmood A. An integrated data warehouse to identify HIV/AIDS prevalence. In: *2024 Horizons of Information Technology and Engineering (HITE)*. IEEE (2024). p. 1–6.

[90] Mishra MK Mohan Trivedi L Farooq M Nimma D Alazzam MB Kiran Bala B. Cybersecurity enhancement in IoT-enabled public health information systems using deep learning techniques. In: *2025 AI-Driven Smart Healthcare for Society 5.0*. IEEE (2025). p. 25–30.

[91] Zhu J. Real-time monitoring for sport and mental health prevention of college student based on wireless sensor network. Prev Med. (2023) 173:107581. 10.1016/j.ypmed.2023.107581 37348766

[92] Zala K Thakkar HK Jadeja R Singh P Kotecha K Shukla M. PRMS: design and development of patients’ e-healthcare records management system for privacy preservation in third party cloud platforms. IEEE Access. (2022) 10:85777–91. 10.1109/ACCESS.2022.3198094

[93] Thantharate P Thantharate A. ZeroTrustBlock: enhancing security, privacy, and interoperability of sensitive data through zerotrust permissioned blockchain. Big Data Cogn Comput. (2023) 7:165. 10.3390/bdcc7040165

[94] Smallwood C Matos C Monteiro H Shapiro M Ngoc MT Elamein M Enhancing information for action: a strategic tool for strengthening public health emergency management systems. Int J Med Inform. (2025) 196:105791. 10.1016/j.ijmedinf.2025.105791 39862567

[95] Ng QA Chiew YS Wang X Tan CP Nor MBM Damanhuri NS Network data acquisition and monitoring system for intensive care mechanical ventilation treatment. IEEE Access. (2021) 9:91859–73. 10.1109/ACCESS.2021.3092194

[96] Ni Y Zhou T Gao X Chen H. Design and implementation of a UPOD for decentralized IoT data platform based on the activitypub protocol. In: *2023 IEEE International Conference on Dependable, Autonomic and Secure Computing, International Conference on Pervasive Intelligence and Computing, International Conference on Cloud and Big Data Computing, International Conference on Cyber Science and Technology Congress (DASC/PiCom/CBDCom/CyberSciTech)*. IEEE (2023). p. 825–30.

[97] Jyothi EV Kranthi M Dankan Gowda V Tanguturi RC. Design of intelligent medical integrity authentication and secure information for public cloud in hospital administration. In: *Proceedings of the 2nd International Conference on Edge Computing and Applications, ICECAA 2023*. Institute of Electrical and Electronics Engineers Inc. (2023). p. 256–61.

[98] Indrasary Y Nugraha D Prihatmanto AS Sarief I Gunawan R Yunautama D. Analysis on performance of semi-real-time transcription on health app. In: *Proceeding of 2024 18th International Conference on Telecommunication Systems, Services, and Applications, TSSA 2024*. Institute of Electrical and Electronics Engineers Inc. (2024).

[99] Park BH Lee S Ozmen O Kumar M Ward M Nebeker JR. Real-time multi-granular analytics framework for HIT systems. In: *2022 IEEE International Conference on Big Data (Big Data)*. IEEE (2022). p. 3441–6.

[100] Zeng D Wu J Yang B Obara T Okawa A Iino N SHECS: a local smart hands-free elderly care support system on smart AR glasses with AI technology. In: *2021 IEEE International Symposium on Multimedia (ISM)*. IEEE (2021). p. 66–74.

[101] Ramu M Harshitha M Hanisha R Chandana P Meghana P. Cardiovascular disease prediction using machine learning classifiers. In: *2023 9th International Conference on Advanced Computing and Communication Systems (ICACCS)*. IEEE (2023). p. 709–15.

[102] Zhou B Yang G Shi Z Ma S. Natural language processing for smart healthcare. IEEE Rev Biomed Eng. (2024) 17:4–18. 10.1109/RBME.2022.3210270 36170385

[103] Dang A Beydoun G. Toward addressing the software architecture blind spot of information system success factors in the public health domain (Tech. rep.). (2023).

[104] Sakib N Jamil SJ Mukta SH. A novel approach on machine learning based data warehousing for intelligent healthcare services. In: *2022 IEEE Region 10 Symposium (TENSYMP)*. IEEE (2022). p. 1–5.

[105] Saraswat D Bhattacharya P Verma A Prasad VK Tanwar S Sharma G Explainable AI for healthcare 5.0: opportunities and challenges. IEEE Access. (2022) 10:84486–517. 10.1109/ACCESS.2022.3197671

[106] Šafran V Horvat S Ilijevec B Roj IR Flis V Mlakar I. Integrating HL7 FHIR into clinical decision support systems: a real-world application with pepper humanoid robot in hospital during doctor visits. In: *2024 9th International Conference on Mathematics and Computers in Sciences and Industry (MCSI)*. IEEE (2024). p. 139–45.

[107] Shanbehzadeh M Kazemi-Arpanahi H Valipour AA Zahedi A. Notifiable diseases interoperable framework toward improving Iran public health surveillance system. J Educ Health Promot. (2021) 10:179. 10.4103/jehp.jehp_1082_20 34250113 PMC8249955

[108] Shihao L Dahnil DP Saad S. A survey of smart campus resource information management in internet of things. IEEE Access. (2025) 13:66622–45. 10.1109/ACCESS.2025.3558900

[109] Sihombing RA Sulistyohati A Paramita A Anggraeni NKP Natsir F Marsiani ES Expert system for early detection of kidney disease through e-health using android-based dempster shafer algorithm. In: *2025 International Conference on Computer Sciences, Engineering, and Technology Innovation (ICoCSETI)*. IEEE (2025). p. 84–9.

[110] Silva FA Nguyen TA Fe I Brito C Min D Lee JW. Performance evaluation of an internet of healthcare things for medical monitoring using M/M/c/K queuing models. IEEE Access. (2021) 9:55271–83. 10.1109/ACCESS.2021.3071508

[111] Zou L Goh HL Liew CJY Quah JL Gu GT Chew JJ Ensemble image explainable AI (XAI) algorithm for severe community-acquired pneumonia and COVID-19 respiratory infections. IEEE Trans Artif Intell. (2023) 4:242–54. 10.1109/TAI.2022.3153754

[112] Souali K Souali M Ouzzif M. An RFID-based traceability approach to improve the overall health status management in Morocco. In: *2023 10th International Conference on Wireless Networks and Mobile Communications (WINCOM)*. IEEE (2023). p. 1–6.

[113] Tang T Nan S Lin L Jin X Liao W Lv X. Why is a rule-based shock early warning system not accurate: a case study. In: *2021 IEEE International Conference on Bioinformatics and Biomedicine (BIBM)*. IEEE (2021). p. 2891–5.

[114] Bossenko I Randmaa R Piho G Ross P. Interoperability of health data using FHIR mapping language: transforming HL7 CDA to FHIR with reusable visual components. Front Digit Health. (2024) 6:1480600. 10.3389/fdgth.2024.1480600 39749099 PMC11693713

[115] Nsaghurwe A Dwivedi V Ndesanjo W Bamsi H Busiga M Nyella E One country’s journey to interoperability: Tanzania’s experience developing and implementing a national health information exchange. BMC Med Inform Decis Mak. (2021) 21:139. 10.1186/s12911-021-01499-6 33926428 PMC8086308

[116] Annie Silviya SH Lakshmi Prabha TS Sunitha T Sriman B Sarran Kumar NK Subin SS. A secure and efficient blockchain-based multi-cloud medical file sharing. In: *2023 International Conference on Advances in Computing, Communication and Applied Informatics (ACCAI)*. IEEE (2023). p. 1–7.

[117] Tania MH Rahman MA Ahmed KU. Medication management application for directly observed treatment of TB patients. In: *2024 6th International Conference on Electrical Engineering and Information & Communication Technology (ICEEICT)*. IEEE (2024). p. 463–8.

[118] Ullah I Alkhalifah A Rehman SU Kumar N Khan MA. An anonymous certificateless signcryption scheme for internet of health things. IEEE Access. (2021) 9:101207–16. 10.1109/ACCESS.2021.3097403

[119] Usharani AV Attigeri G. Secure EMR classification and deduplication using mapreduce. IEEE Access. (2022) 10:34404–14. 10.1109/ACCESS.2022.3161439

[120] Vasileiou N Giannakopoulou O Manta O Bromis K Vagenas TP Kouris I FHIR-driven advancements in healthcare interoperability: insights from the retention project. In: *2024 IEEE International Conference on Engineering, Technology, and Innovation (ICE/ITMC)*. IEEE (2024). p. 1–6.

[121] Tan AL Feliciano EJG Habaluyas KER Bolong NP Wong JQ. Processes of creating a national cancer registry system in the Philippines. Electron J Inform Syst Dev Ctries. (2024) 90:e12324. 10.1002/isd2.12324

[122] Wang W Khalajzadeh H Grundy J Madugalla A Obie HO. Adaptive user interfaces for software supporting chronic disease. In: *Proceedings of the 46th International Conference on Software Engineering: Software Engineering in Society*. New York, NY, USA: ACM (2024). p. 118–29.

[123] Alias SA Mohd Ali D Yusuf Y. Internet of things based heart failure monitoring system using radio frequency identification. In: *19th IEEE Student Conference on Research and Development: Sustainable Engineering and Technology Towards Industry Revolution, SCOReD 2021*. Institute of Electrical and Electronics Engineers Inc. (2021). p. 96–101.

[124] Anitha P Priya R. A new hybrid EAES-SI framework for secure information sharing iin WBANS. In: *7th International Conference on Electronics, Communication and Aerospace Technology, ICECA 2023 – Proceedings*. Institute of Electrical and Electronics Engineers Inc. (2023). p. 1698–705.

[125] Wu H Pan Y Weng X Chen H. Design of campus health information system using face recognition and body temperature detection. In: *2021 IEEE International Conference on Dependable, Autonomic and Secure Computing, International Conference on Pervasive Intelligence and Computing, International Conference on Cloud and Big Data Computing, International Conference on Cyber Science and Technology Congress (DASC/PiCom/CBDCom/CyberSciTech)*. IEEE (2021). p. 873–8. 10.1109/DASC-PICom-DataCom-CyberSciTec.2017.201 PMC6157906 30272054

[126] Aruna A Sellam V. Security framework for electronic health record in the cloud based on public key encryption. In: *2023 9th International Conference on Signal Processing and Communication, ICSC 2023*. Institute of Electrical and Electronics Engineers Inc. (2023). p. 135–8.

[127] Leygonie R Vimont G. Empirical analysis of the residual information needed for image classification. In: *2021 IEEE International Conference on Big Data (Big Data)*. IEEE (2021). p. 2813–7.

[128] Bodin ON Bezborodova OE Mitroshin AN Chuvykin BV Martinov DV Edemsky MV. Optimization of medical care provision in intelligent medical information system. In: *Proceedings of the 2023 International Conference on Systems and Technologies of the Digital HealthCare, STDH 2023*. Institute of Electrical and Electronics Engineers Inc. (2023). p. 149–52.

[129] Veikkolainen P Tuovinen T Kulmala P Jarva E Juntunen J Tuomikoski AM The evolution of medical student competencies and attitudes in digital health between 2016 and 2022: comparative cross-sectional study. JMIR Med Educ. (2025) 11:e67423. 10.2196/67423 40743520 PMC12313084

[130] Messai A Drif A Ouyahia A Guechi M Rais M Kaderali L Transparent AI models for meningococcal meningitis diagnosis: evaluating interpretability and performance metrics. In: *International IEEE Conference Proceedings, IS*. Institute of Electrical and Electronics Engineers Inc. (2024).

[131] Kalita KP Chettri SK Deka RK. A blockchain-based model for maternal health information exchange and prediction of health risks using machine learning. In: *Proceedings of the International Conference on Intelligent and Innovative Technologies in Computing, Electrical and Electronics, ICIITCEE 2023*. Institute of Electrical and Electronics Engineers Inc. (2023). p. 1184–9.

[132] Ricci A Croatti A Montagna S. Pervasive and connected digital twins—a vision for digital health. IEEE Internet Comput. (2022) 26:26–32. 10.1109/MIC.2021.3052039.

[133] López-Úbeda P Carlos Díaz-Galiano M Alfonso Ureña-López L Teresa Martín-Valdivia M. Pre-trained language models to extract information from radiological reports (Tech. rep.). (2021).

[134] Snegireva E Khazankin GR Mikheenko I. System architecture for reading and interpreting physical printouts of medical forms. In: *International Conference of Young Specialists on Micro/Nanotechnologies and Electron Devices, EDM*. IEEE Computer Society (2021). p. 547–50.

[135] Murat B Uzer AO Ketenci S Yasbek S Korkmaz I. A symptom evaluation system on medical diagnosis. In: *TIPTEKNO 2023 – Medical Technologies Congress, Proceedings*. Institute of Electrical and Electronics Engineers Inc. (2023).

[136] Kumar V Sarkar A Jana A. Big data and WebGIS for formulating health care policy in India. In: *2021 IEEE Conference on Norbert Wiener in the 21st Century: Being Human in a Global Village, 21CW 2021*. Institute of Electrical and Electronics Engineers Inc. (2021).

[137] Nabasirye A Ssali IW. Integrating Natural Language Processing and Large Language Models into DHIS2 to Improve Health Data Utilization. Ottawa: Institute of Electrical and Electronics Engineers (IEEE) (2025). p. 47–52.

[138] Nazakat M Khalique F Khan SA Ahsan N. Towards data driven spatio-temporal threshold identification based on cost effective public health information management framework. IEEE Access. (2022) 10:16634–43. 10.1109/ACCESS.2022.3149349

[139] Arunachalam S Shanthi HJ Sivagurunathan G Das S Anand D Raj MT. Cloud-based decentralized smart healthcare for patient monitoring on deep learning. In: *Proceedings of the 2nd International Conference on Applied Artificial Intelligence and Computing, ICAAIC 2023*. Institute of Electrical and Electronics Engineers Inc. (2023). p. 459–66.

[140] Lindgren H Kampik T Rosero EG Blusi M Nieves JC. Argumentation-based health information systems: a design methodology. IEEE Intell Syst. (2021) 36:72–80. 10.1109/MIS.2020.3044944.

[141] Chaitra R Dhananjaya V. An enhanced mechanism for securing patient vital health information in public cloud. In: *Proceedings of the 2023 2nd International Conference on Augmented Intelligence and Sustainable Systems, ICAISS 2023*. Institute of Electrical and Electronics Engineers Inc. (2023). p. 1323–8.

[142] Marinescu IA Rotaru CM Nicolau D Krawiec P. Challenges and perspectives for the development of a future ecosystem for elderly within pandemic. In: *Proceedings—2021 23rd International Conference on Control Systems and Computer Science Technologies, CSCS 2021*. Institute of Electrical and Electronics Engineers Inc. (2021). p. 501–8.

[143] Ivanov IE Ivanov B. Unified national digital framework for exchange and storage of medical image information. In: *2022 10th International Scientific Conference on Computer Science, COMSCI 2022—Proceedings*. Institute of Electrical and Electronics Engineers Inc. (2022).

[144] Benbrahim H Hachimi H Amine A. The Moroccan health data bank: a proposal for a national electronic health system based on big data. J Inform Technol Manage. (2024) 16:79–97. 10.22059/jitm.2024.96376

[145] Gayathri S Jaya Mabel Rani A Anushalin PS Ravi CN Jeyalaxmi M Samuthira Pandi V. A novel approach to design secured and privacy enabled health data linkage system based on cyber security principles. In: *2024 IEEE International Conference on Big Data & Machine Learning (ICBDML)*. IEEE (2024). p. 14–20.

[146] Mutasa L Ujakpa MM Nyikana W Shaanika I Iyamu T. Application of enterprise architecture to guide the integration of health information systems in Namibia. Inform Resour Manage J. (2025) 38:1–22. 10.4018/IRMJ.367274

[147] Komal T Kandasamy S Meenalakshmi S Devi RM. Blockchain based effective ledger and decentralization in healthcare system. In: *6th International Conference on Electronics, Communication and Aerospace Technology, ICECA 2022—Proceedings*. Institute of Electrical and Electronics Engineers Inc. (2022). p. 736–42.

[148] Sundararaman A. Data platform to accelerate healthcare insights generation. In: *Proceedings—2023 IEEE 11th International Conference on Healthcare Informatics, ICHI 2023*. Institute of Electrical and Electronics Engineers Inc. (2023). p. 470–5.

[149] Winter A Jahn F Löbe M Stäubert S. The European health data space as 3LGM2-based enterprise architecture model. Stud Health Technol Inform. (2025) 327:647–51. 10.3233/SHTI250428 40380537

[150] Anastasiadou MN Isaia P Kolios P Eliades DG Laoudias C. Leveraging ICTs for effective COVID-19 pandemics management: insights from a health information system implementation in cyprus. In: *Proceedings—2024 IEEE/ACM 24th International Symposium on Cluster, Cloud and Internet Computing Workshops, CCGridW 2024*. Institute of Electrical and Electronics Engineers Inc. (2024). p. 84–91.

[151] Qureshi NS Midhun Chakkaravarthy D Jamal N. Domestic intranet cloud design for connecting district health information systems for capacity development of healthcare in developing countries. In: *1st International Conference on Innovative Engineering Sciences and Technological Research, ICIESTR 2024—Proceedings*. Institute of Electrical and Electronics Engineers Inc. (2024).

[152] Olca E Can O. DICON: a domain-independent consent management for personal data protection. IEEE Access. (2022) 10:95479–97. 10.1109/ACCESS.2022.3204970

[153] Bandi A Fellah A. An implementation and evaluation of blockchain-based digital health passports. In: *5th International Conference on Inventive Computation Technologies, ICICT 2022—Proceedings*. Institute of Electrical and Electronics Engineers Inc. (2022). p. 476–82.

[154] Biswas S Sharif K Li F Alam I Mohanty SP. DAAC: digital asset access control in a unified blockchain based e-health system. IEEE Trans Big Data. (2022) 8:1273–87. 10.1109/TBDATA.2020.3037914

[155] Swami Das M Kumar GR Lakshmi DV. Healthcare mobile app for rural areas and recent advancements. In: *5th IEEE International Conference on Cybernetics, Cognition and Machine Learning Applications, ICCCMLA 2023*. Institute of Electrical and Electronics Engineers Inc. (2023). p. 209–20.

[156] Rojo J Garcia-Alonso J Berrocal J Foschini L Bellavista P Hernandez J Blockchains’ federation: developing personal health trajectory-centered health systems. In: *Proceedings – 23rd IEEE/ACM International Symposium on Cluster, Cloud and Internet Computing Workshops, CCGridW 2023*. Institute of Electrical and Electronics Engineers Inc. (2023). p. 81–8.

[157] Gamal A Barakat S Rezk A. Integrated document-based electronic health records persistence framework. Int J Adv Comput Sci Appl. (2021) 12:2021. 10.14569/IJACSA.2021.0121017

[158] Diaz AG Gumtang AD Orpiada CJA Balagot AS Villanueva EA Manalang MA. PHIrecord: a medical record management system for rural health facilities in the Philippines. In: *2024 6th IEEE Symposium on Computers and Informatics, ISCI 2024*. Institute of Electrical and Electronics Engineers Inc. (2024). p. 188–93.

[159] Ali Saberi M McHeick H Adda M Ibrahim H. Toward implementing interoperability in pervasive healthcare systems for chronic diseases by decentralization and modularity. In: *2022 3rd International Conference on Human-Centric Smart Environments for Health and Well-Being, IHSH 2022*. Institute of Electrical and Electronics Engineers Inc. (2022). p. 64–72.

[160] Tummers J Tobi H Catal C Tekinerdogan B Schalk B Leusink G. A health information systems architecture study in intellectual disability care: commonalities and variabilities. Healthc Anal. (2024) 5:100295. 10.1016/j.health.2023.100295

[161] Araujo A De Barros Vidal F Martins ACF Bonifacio R Atique M Fernandes JHC. An overview of the information systems in primary care of the Brazil’s unified health system. In: *2022 17th Iberian Conference on Information Systems and Technologies (CISTI)*. IEEE (2022). p. 1–6.

[162] Yongjoh S So-In C Kompunt P Muneesawang P Morien RI. Development of an internet-of-healthcare system using blockchain. IEEE Access. (2021) 9:113017–31. 10.1109/ACCESS.2021.3103443

[163] H aliima N Rushingabigwi G Nzanywayingoma F. Design of an IoT based monitoring system for expectant rural women in developing countries. In: *Proceedings of the 2nd 2022 International Conference on Computer Science and Software Engineering, CSASE 2022*. Institute of Electrical and Electronics Engineers Inc. (2022). p. 41–7.

[164] Rajasekharan A Koshy R. EMRChain: electronic medical records management system using blockchain. In: *2024 IEEE International Conference on Blockchain and Distributed Systems Security, ICBDS 2024*. Institute of Electrical and Electronics Engineers Inc. (2024).

[165] Tummers J Tobi H Catal C Tekinerdogan B. Designing a reference architecture for health information systems. BMC Med Inform Decis Mak. (2021) 21:210. 10.1186/s12911-021-01570-2 34238281 PMC8263849

[166] Perbix M Löbe M Stäubert S Anil Sinaci A Gencturk M Quintero M A formal model for the FAIR4Health information architecture. In: Mantas J, Gallos P, Zoulias E, Hasman A, Househ MS, Diomidous M, et al., editors. *Studies in Health Technology and Informatics*. Berlin: IOS Press BV (2022). Vol. 295. p. 446–9. 10.3233/SHTI220761 35773907

[167] Fragidis L Tsamoglou S Kosmidis K Aggelidis V. Architectural design of national evidence based medicine information system based on electronic health record. Technol Health Care. (2024) 32:4187–201. 10.3233/THC-232042. 39031405 PMC11613116

[168] Guimaraes T Duarte R Cunha J Gomes P Santos MF. Security and immutability of open data in healthcare. In: Shakshuki E, editors. *Procedia Computer Science*. Leuven: Elsevier B.V. (2023). Vol. 220. p. 832–7.

[169] He W Zhang JZ Wu H Li W Shetty S. A unified health information system framework for connecting data, people, devices, and systems. J Glob Inform Manage. (2022) 30:1–30. 10.4018/JGIM.305239

[170] Surasak T. Blockchain-enhanced security and efficiency for Thailand’s health information system (Tech. Rep. 11). (2024).

[171] Thamrin A Xu H. Hierarchical cloud-based consortium blockchains for healthcare data storage. In: *Proceedings—2021 21st International Conference on Software Quality, Reliability and Security Companion, QRS-C 2021*. Institute of Electrical and Electronics Engineers Inc. (2021). p. 644–51.

[172] Osei-Tutu K Song YT. A microservices enterprise architecture for healthcare information exchange (HIE) in developing countries. In: *Proceedings—2021 10th International Congress on Advanced Applied Informatics, IIAI-AAI 2021*. Institute of Electrical and Electronics Engineers Inc. (2021). p. 762–7.

[173] Marques C Ramos V Peixoto H Machado J. Pervasive monitoring system for services and servers in healthcare environment. In: Shakshuki E, editors. *Procedia Computer Science*. Porto: Elsevier B.V. (2022). Vol. 201. p. 720–5.

[174] Aryanto KYE Seputra KA Wijaya INSW Abyong IW Pradnyana GA Paramartha AAY. Towards healthcare data sharing: an e-health integration effort in Indonesian district. In: *Proceedings—2021 International Seminar on Application for Technology of Information and Communication: IT Opportunities and Creativities for Digital Innovation and Communication within Global Pandemic, iSemantic 2021*. Institute of Electrical and Electronics Engineers Inc. (2021). p. 280–4.

[175] Bazel MA Ahmad M Mohammed F. Hospital information systems in Malaysia: current issues and blockchain technology as a solution. In: *2022 2nd International Conference on Emerging Smart Technologies and Applications, eSmarTA 2022*. Institute of Electrical and Electronics Engineers Inc. (2022).

[176] Klementi T Piho G Ross P. A reference architecture for personal health data spaces using decentralized content-addressable storage networks. Front Med. (2024) 11:1411013. 10.3389/fmed.2024.1411013 PMC11286498 39081693

[177] Rwegasira D Kimaro H Mbiaji R Julius J Minde V Mussa B Deployment and innovation processes of integrated electronic medical record (EMR) system: a case of university health centre living lab in Tanzania. In: *2024 IST-Africa Conference (IST-Africa)*. IEEE (2024). p. 1–8.

[178] Gazzarata R Maggi N Magnoni LD Monteverde ME Ruggiero C Giacomini M. Semantics management for a regional health information system in Italy by CTS2 and FHIR. In: Delgado J, Benis A, de Toledo P, Gallos P, Giacomini M, Martínez-García A, et al., editors. *Studies in Health Technology and Informatics*. Berlin: IOS Press BV (2021). Vol. 287. p. 119–23. 10.3233/SHTI210828 34795094

[179] Rinty MR Prodhan UK Rahman MM. A prospective interoperable distributed e-Health system with loose coupling in improving healthcare services for developing countries. Array. (2022) 13:100114. 10.1016/j.array.2021.100114

[180] Hoque ASML Mia MR Abdullah MS Islam MJ Nath BCD Rahman MT BlockPRLS: blockchain-based patient record linkage system for big data analytics. In: *Proceedings—2024 IEEE 48th Annual Computers, Software, and Applications Conference, COMPSAC 2024*. Institute of Electrical and Electronics Engineers Inc. (2024). p. 877–86.

[181] Dowden JJ Pretty RW Shea JM Dermody M Doyle G Antle S A novel technology for harmonizing and analyzing cancer data. Observations from integrating health connect in Newfoundland and Labrador, Canada. Health Inform J. (2024) 30:14604582241267792. 10.1177/14604582241267792 39056109

[182] Tsegaye T Flowerday S. A system architecture for ensuring interoperability in a South African national electronic health record system. South Afr Comput J. (2021) 33:79–110. 10.18489/sacj.v33i1.838

[183] Wijayanti D Urbaya S Sitompul T Adrian V. E-government interoperability: provincial-level architecture model to enable fast healthcare interoperability resources (FHIR). In: *Proceedings of 2024 International Conference on Information Management and Technology, ICIMTech 2024*. Institute of Electrical and Electronics Engineers Inc. (2024). p. 811–6.

[184] Kastowo D Utami E Hendi Muhammad A. FHIR, BigchainDB, and GraphQL approach for interoperability between heterogeneous health information system. In: *ICOIACT 2022—5th International Conference on Information and Communications Technology: A New Way to Make AI Useful for Everyone in the New Normal Era, Proceeding*. Institute of Electrical and Electronics Engineers Inc. (2022). p. 272–7.

[185] Koren A Prasad R. Setting standards for personal health data in the age of 5G and 6G networks. J ICT Stand. (2024) 12:47–70. 10.13052/jicts2245-800X.1213

[186] Shravan M Sanjay HA Shastry KA Rithesh Ramesh D Hemant V Laxman K. Interoperability in blockchain based healthcare. In: *MysuruCon 2022—2022 IEEE 2nd Mysore Sub Section International Conference*. Institute of Electrical and Electronics Engineers Inc. (2022).

[187] Mammadova M Ahmadova A. Formation of unified digital health information space in healthcare 4.0 environment and interoperability issues. In: *16th IEEE International Conference on Application of Information and Communication Technologies, AICT 2022—Proceedings*. Institute of Electrical and Electronics Engineers Inc. (2022).

[188] De Sousa OVJ Coutinho C. Interoperability between information systems concerning electronic records of patients. In: *2022 International Symposium on Sensing and Instrumentation in 5G and IoT Era, ISSI 2022*. Institute of Electrical and Electronics Engineers Inc. (2022). p. 121–6.

[189] Aborujilah A Elsebaie AEFM Mokhtar SA. IoT MEMS: IoT-based paradigm for medical equipment management systems of ICUs in light of COVID-19 outbreak. IEEE Access. (2021) 9:131120–33. 10.1109/ACCESS.2021.3069255 34786319 PMC8545208

[190] Lindquist W Helal S Khaled A Hutchinson W. IoTility: architectural requirements for enabling health IoT ecosystems. IEEE Trans Emerg Top Comput. (2021) 9:1206–18. 10.1109/TETC.2019.2957241.

[191] Mishra R Prasad R. Towards efficient and secure framework for devices and informatics for internet of medical things. In: *International Symposium on Wireless Personal Multimedia Communications, WPMC*. IEEE Computer Society (2022). p. 459–63.

[192] Samonte MJC Magno FDC Ang KGY Lalap KEM. AidConnect: a telehealth web-based system with smartwatch integration for enhanced medical tracking support in aged care facilities for caregivers of elderly individuals in the Philippines. In: *Proceedings—2024 13th International Conference on Computer Technologies and Development, TechDev 2024*. Institute of Electrical and Electronics Engineers Inc. (2024). p. 96–102.

[193] Vellela SS Reddy VL Roja D Rao GR Khader Basha SK Kumar KK. A cloud-based smart IoT platform for personalized healthcare data gathering and monitoring system. In: *2023 3rd Asian Conference on Innovation in Technology, ASIANCON 2023*. Institute of Electrical and Electronics Engineers Inc. (2023).

[194] Cheng D Fu Y Zhou Y. Basic medical information sharing system design based on IoT technology. In: *Proceedings—2022 International Conference on Intelligent Transportation, Big Data and Smart City, ICITBS 2022*. Institute of Electrical and Electronics Engineers Inc. (2022). p. 375–8.

[195] Zampognaro P Paragliola G Falanga V. A FHIR based architecture of a multiprotocol IoT home gateway supporting dynamic plug of new devices within instrumented environments. In: *Proceedings – IEEE Symposium on Computers and Communications*. Institute of Electrical and Electronics Engineers Inc. (2021).

[196] Koren A Jurcevic M Prasad R. Semantic constraints specification and schematron-based validation for internet of medical things- data. IEEE Access. (2022) 10:65658–70. 10.1109/ACCESS.2022.3182486

[197] Ahmed KR Islam R Alam MA Rivin MAH Alam M Rahman MS. A management information systems framework for sustainable cloud-based smart e-healthcare research information systems in Bangladesh. In: *2024 Asian Conference on Intelligent Technologies, ACOIT 2024*. Institute of Electrical and Electronics Engineers Inc. (2024).

[198] Zhang W Ming Y Li G. Research on application of computer big data and mobile communication technology in intelligent nursing service platform. In: *2022 IEEE International Conference on Electrical Engineering, Big Data and Algorithms, EEBDA 2022*. Institute of Electrical and Electronics Engineers Inc. (2022). p. 410–4.

[199] Debauche O Nkamla Penka JB Mahmoudi S Lessage X Hani M Manneback P RAMi: a new real-time internet of medical things architecture for elderly patient monitoring. Information. (2022) 13:423. 10.3390/info13090423

[200] Biswas S Sharif K Li F Bairagi AK Latif Z Mohanty SP. GlobeChain: an interoperable blockchain for global sharing of healthcare data—a COVID-19 perspective. IEEE Consum Electron Mag. (2021) 10:64–9. 10.1109/MCE.2021.3074688.

[201] Kumari T Kumari R Devi N Sharma B. Personalized healthcare monitoring system. In: *Proceedings of the 5th International Conference on Trends in Electronics and Informatics, ICOEI 2021*. Institute of Electrical and Electronics Engineers Inc. (2021). p. 1088–96.

[202] Zhang K Gao Q Zhang J Dai D Han Y Zan H Construction of Chinese pediatric epilepsy knowledge graph. In: *2023 IEEE 36th International Symposium on Computer-Based Medical Systems (CBMS)*. IEEE (2023). p. 241–4.

[203] Piamjinda P Boonnag C Ittichaiwong P Rattanasonrerk S Veerakanjana K Duangchaemkarn K CHIVID: a rapid deployment of community and home isolation during COVID-19 pandemics. IEEE J Transl Eng Health Med. (2024) 12:390–400. 10.1109/JTEHM.2024.3377258 38606388 PMC11008800

[204] Hu L Kar P. Design of a blockchain-based secure health monitoring system using decentralized machine learning technique. IEEE Commun Mag. (2024) 62:46–52. 10.1109/MCOM.002.2300610

[205] Aleburu D Mabude C Mustapha A Umoren U Kuyoro S. HINE-block: blockchain based model for secure health information exchange. In: *International Conference on Science, Engineering and Business for Driving Sustainable Development Goals, SEB4SDG 2024*. Institute of Electrical and Electronics Engineers Inc. (2024).

[206] Malik R Ur-Rehaman A Razzaq H Bhatt C Kaushik K Khan IU. Advancing healthcare IoT: blockchain and federated learning integration for enhanced security and insights. In: *Proceedings of International Conference on Communication, Computer Sciences and Engineering, IC3SE 2024*. Institute of Electrical and Electronics Engineers Inc. (2024). p. 308–14.

[207] Manoharan J Ali AH Aljohani MM Soni A Gowrishankar V Upadhyay S. Experimental possibilities of decentralized health information system interoperability using blockchain technology. In: *5th International Conference on Sustainable Communication Networks and Application, ICSCNA 2024—Proceedings*. Institute of Electrical and Electronics Engineers Inc. (2024). p. 432–8.

[208] Haritha T Anitha A. Multi-level security in healthcare by integrating lattice-based access control and blockchain-based smart contracts system. IEEE Access. (2023) 11:114322–40. 10.1109/ACCESS.2023.3324740

[209] Diaz A Kaschel H. Scalable management architecture for electronic health records based on blockchain. In: *2022 IEEE International Conference on Automation/25th Congress of the Chilean Association of Automatic Control: For the Development of Sustainable Agricultural Systems, ICA-ACCA 2022*. Institute of Electrical and Electronics Engineers Inc. (2022).

[210] Amaracitra DS Alamsyah A. Decentralized Medical Record Model Using Composable NFT: Achieving Data Security, Interoperability, and Privacy through Granular Data Access. Cairo: Institute of Electrical and Electronics Engineers (IEEE) (2025). p. 1–6.

[211] Kumhar M Bhatia J Jadav NK Gupta R Tanwar S. AI-based intelligent SDN controller to optimize onion routing framework for IoMT environment. In: *2023 IEEE International Conference on Communications Workshops: Sustainable Communications for Renaissance, ICC Workshops 2023*. Institute of Electrical and Electronics Engineers Inc. (2023). p. 885–90.

[212] Hasselgren A Rensaa JAH Kralevska K Gligoroski D Faxvaag A. Blockchain for increased trust in virtual health care: proof-of-concept study. J Med Internet Res. (2021) 23:e28496. 10.2196/28496 34328437 PMC8367164

[213] Al-Aswad H El-Medany WM Balakrishna C Ababneh N Curran K. BZKP: blockchain-based zero-knowledge proof model for enhancing healthcare security in Bahrain IoT smart cities and COVID-19 risk mitigation. Arab J Basic Appl Sci. (2021) 28:154–71. 10.1080/25765299.2020.1870812

[214] Lee S Kim S. Blockchain as a cyber defense: opportunities, applications, and challenges. IEEE Access. (2022) 10:2602–18. 10.1109/ACCESS.2021.3136328

[215] Zao JKK Wu JTS Kanyimbo K Delizy F Gan TT Kuo HI Design of a trustworthy cloud-native national digital health information infrastructure for secure data management and use. Oxf Open Digit Health. (2024) 2:oqae043. 10.1093/oodh/oqae043 40230982 PMC11932406

[216] Kumar J Ali AS Kumar A Kumar S. Enhancing patient data security through blockchain adoption in Fiji’s healthcare system. In: *Proceedings of 2023 International Conference on Sustainable Technology and Engineering, i-COSTE 2023*. Institute of Electrical and Electronics Engineers Inc. (2023).

[217] Richards M. Software Architecture Patterns. 2nd ed. Sebastopol: O’Reilly (2015). p. 7–44.

[218] Weng X Wu H Pan Y Chen H. Decentralized personal cloud data model and its application in campus health information system. In: *2021 IEEE International Conference on Dependable, Autonomic and Secure Computing, International Conference on Pervasive Intelligence and Computing, International Conference on Cloud and Big Data Computing, International Conference on Cyber Science and Technology Congress (DASC/PiCom/CBDCom/CyberSciTech)*. IEEE (2021). p. 879–83.

[219] Wu D Wang Y. Revolutionizing healthcare information systems with blockchain. Front Digit Health. (2024) 5:1329196. 10.3389/fdgth.2023.1329196 38274085 PMC10808696

[220] Shukla M Lin J Seneviratne O. BlockIoT-RETEL: blockchain and iot based read-execute-transact-erase-loop environment for integrating personal health data. In: *Proceedings—2021 IEEE International Conference on Blockchain, Blockchain 2021*. Institute of Electrical and Electronics Engineers Inc. (2021). p. 237–43.

[221] Yeung AWK Litvinova O Bragazzi NL Khader Y Rahman MM Said Z Digital health and mobile health: a bibliometric analysis of the 100 most cited papers and their contributing authors. In: Gong Z, editors. *Exploration of Digital Health Technologies*. New York: Open Exploration Publishing Inc. (2024). p. 86–100.

[222] Ayorinde AA Williams I Mannion R Song F Skrybant M Lilford RJ Publication and related biases in health services research: a systematic review of empirical evidence. BMC Med Res Methodol. (2020) 20:137. 10.1186/s12874-020-01010-1 32487022 PMC7268600

[223] Ayorinde AA Williams I Mannion R Song F Skrybant M Lilford RJ Assessment of publication bias and outcome reporting bias in systematic reviews of health services and delivery research: a meta-epidemiological study. PLoS One. (2020) 15:e0227580. 10.1371/journal.pone.0227580 31999702 PMC6992172

[224] Martins D Lewerenz S Carmo A Martins H. Interoperability of telemonitoring data in digital health solutions: a scoping review. Front Digit Health. (2025) 7:1502260. 10.3389/fdgth.2025.1502260 40330872 PMC12052697

[225] Haddad T Kumarapeli P de Lusignan S Barman S Khaddaj S. A sustainable future in digital health: leveraging environmentally friendly architectural tactics for sustainable data processing. Stud Health Technol Inform. (2025) 327:713–7. 10.3233/SHTI250441 40380550

[226] Bathelt F Lorenz S Weidner J Sedlmayr M Reinecke I. Application of modular architectures in the medical domain – a scoping review. J Med Syst. (2025) 49:27. 10.1007/s10916-025-02158-3 39964566 PMC11835905

[227] Liu X Rivera SC Moher D Calvert MJ Denniston AK. Reporting guidelines for clinical trial reports for interventions involving artificial intelligence: the CONSORT-AI Extension. BMJ. (2020) 370:m3164. 10.1136/bmj.m3164 32909959 PMC7490784

[228] Rivera SC Liu X Chan AW Denniston AK Calvert MJ. Guidelines for clinical trial protocols for interventions involving artificial intelligence: the SPIRIT-AI Extension. BMJ. (2020) 370:m3210. 10.1136/bmj.m3210 32907797 PMC7490785

[229] Facile R Chronaki C van Reusel P Kush R. Standards in sync: five principles to achieve semantic interoperability for TRUE research for healthcare. Front Digit Health. (2025) 7:1567624. 10.3389/fdgth.2025.1567624 40538570 PMC12176726

[230] Rahmani AM Babaei Z Souri A. Event-driven IoT architecture for data analysis of reliable healthcare application using complex event processing. Cluster Comput. (2021) 24:1347–60. 10.1007/s10586-020-03189-w

[231] Shaikh N Kasat K Godi RK Krishna VR Chauhan DK Kharade J. Novel IoT framework for event processing in healthcare applications. Meas Sens. (2023) 27:100733. 10.1016/j.measen.2023.100733

[232] Wagholikar KB Mandel JC Klann JG Wattanasin N Mendis M Chute CG SMART-on-FHIR implemented over i2b2. J Am Med Inform Assoc. (2017) 24:398–402. 10.1093/jamia/ocw079 27274012 PMC5391721

[233] Carlos Ferreira J Elvas LB Correia R Mascarenhas M. Enhancing EHR interoperability and security through distributed ledger technology: a review. Healthcare. (2024) 12:1967. 10.3390/healthcare12191967 39408147 PMC11477175

[234] Mandel JC Kreda DA Mandl KD Kohane IS Ramoni RB. SMART on FHIR: a standards-based, interoperable apps platform for electronic health records. J Am Med Inform Assoc. (2016) 23:899–908. 10.1093/jamia/ocv189 26911829 PMC4997036

