# Supplementary material for: Architectural patterns for health information systems: a systematic review
Source: Front Digit Health. 2025 Nov 17;7:1694839. doi: 10.3389/fdgth.2025.1694839 (PMC12666560; doi:10.3389/fdgth.2025.1694839)
Supplement: Supplementary file 1 [file Datasheet1.pdf]

Supplementary Material

1 PRISMA 2020 COMPLIANCE CHECKLIST

The review was conducted and reported in accordance with the Preferred Reporting Items for Systematic Reviews and Meta-Aanlyses (PRISMA) 2020 statement. The completed PRISMA 2020 checklist summarizes the degree of compliance with each item across the title, abstract, methods, results, discussion, and other information sections. All methodological details, (elegibility criteria, search strategies, data extraction procedures, and risk-of-bias assessment), are fully described in the manuscript and supplementary materials.

Section/Topic	Item	Checklist Item	Reported (Yes/No)	Obs
**TITLE**	1	Identify the report as a systematic review.	Yes	Title identifies the work as a systematic review of software architectures in digital health.
**ABSTRACT**	2	Provide a structured summary.	Yes	Structured abstract includes background, objectives, methods, eligibility, results, and conclusions.
**INTRODUCTION**	3	Describe the rationale for the review.	Yes	Introduction explains the research gap between health information systems and software engineering approaches.
**INTRODUCTION**	4	Provide explicit objectives or research questions.	Yes	Objectives clearly stated to characterize software architecture practices in digital health systems.
**METHODS**	5	Specify inclusion and exclusion criteria.	Yes	Eligibility criteria defined separately for abstract and full-text screening in Supplementary Material Risk of Bias Assessment section.
**METHODS**	6	Specify information sources.	Yes	Four databases: IEEE Xplore, Scopus, PubMed, and Web of Science, search period 2020–2025.
**METHODS**	7	Present search strategies.	Yes	Full search strings for each database in Supplementary Material Search Strings section.

Section/Topic	Item	Checklist Item	Reported (Yes/No)	Obs
**METHODS**	8	Screening process.	Yes	Titles and abstracts screened using eligibility criteria; full-text inclusion performed according to criteria in Supplementary Material.
**METHODS**	9	Data collection process.	Yes	Data extracted for study characteristics, architectural elements, and context description; manually curated from included studies.
**METHODS**	10	List and define variables.	Yes	Variables include study type, architectural style, context, and evaluated features.
**METHODS**	11	Risk of bias assessment.	Yes	Conducted using adapted JBI dimensions; see Risk of Bias Assessment section.
**METHODS**	12	Synthesis methods.	Yes	Narrative synthesis integrating thematic and architectural dimensions; described in Analysis Plan section.
**METHODS**	13	Explore heterogeneity.	Yes	Qualitative heterogeneity addressed through comparison of architectural styles and domain contexts.
**METHODS**	14	Sensitivity analysis.	No	Not applicable; narrative synthesis, no quantitative sensitivity analysis conducted.
**METHODS**	15	Certainty assessment.	Yes	Confidence derived from methodological quality and bias evaluation.
**RESULTS**	16	Search and selection results.	Yes	PRISMA flow diagram included; search and inclusion counts described in Results section.
**RESULTS**	17	Excluded studies and reasons.	Yes	Reasons for exclusion described in Methods; summary of screening included in Supplementary Materials.
**RESULTS**	18	Characteristics of included studies.	Yes	Detailed for 89 studies; identifiers and key attributes presented.

Section/Topic	Item	Checklist Item	Reported (Yes/No)	Obs
**RESULTS**	19	Risk-of-bias results.	Yes	Risk-of-bias matrix in Supplementary Materials Risk of Bias section summarizing all studies.
**RESULTS**	20	Results of individual syntheses.	Yes	Narrative synthesis of architectural characteristics and health domains provided.
**RESULTS**	21	Heterogeneity investigations.	Yes	Addressed through thematic grouping of architectural patterns and design paradigms.
**RESULTS**	22	Sensitivity analyses.	No	Not performed; qualitative synthesis only.
**DISCUSSION**	23	General interpretation.	Yes	Findings interpreted within context of current architectural and informatics research.
**DISCUSSION**	24	Limitations of evidence.	Yes	Limitations of included studies acknowledged in Discussion.
**DISCUSSION**	25	Limitations of review processes.	Yes	Limitations of search scope and inclusion period discussed.
**OTHER INFORMATION**	26	Registration and protocol.	Yes	Not registered; described in methodology.
**OTHER INFORMATION**	27	Support and funding.	Yes	No external funding; institutional support acknowledged.

Table S1: Completed PRISMA 2020 checklist for the present systematic review, summarizing the reporting compliance of all 27 items according to Page et al. [2020].

2 SEARCH STRINGS

Search strategies combined terms related to health information systems and software engineering. Queries were adapted to the syntax of each database, and are shown in Table S2. Searches were limited to the period 2020-2025.

Database	Search string
IEEE Xplore	('All Fulltext and Metadata':'health information system' and 'architecture')

Database	Search string
Scopus	TITLE-ABS-KEY ('health information system' AND 'architecture') AND (LIMIT-TO (PUBYEAR , 2021) OR LIMIT-TO (PUBYEAR , 2022) OR LIMIT-TO (PUBYEAR , 2023) OR LIMIT-TO (PUBYEAR , 2024) OR LIMIT-TO (PUBYEAR , 2025))
PubMed	('health information system' and 'architecture') AND (('2021/01/01'[Date - Publication] : '3000'[Date - Publication]))
Web of Science	(ALL=('health information system' and 'architecture')) AND PY=(2021-2025)

Table S2: Search strings used

3 RISK OF BIAS ASSESSMENT

A structured risk-of-bias assessment was performed to evaluate the methodological rigor and credibility of the studies included in this review. The appraisal followed qualitative evaluation principles derived from the Joanna Briggs Institute (JBI) critical appraisal tools, which were adapted for evaluation of a design-science and software-architecture research within the scope of digital health.

Each study was independently appraised across dimensions: (C1). Alignment between research objective and study design(C2). Clarity of the context and representation of the phenomenon of interest(C3). Appropriateness of data collection and analysis methods(C4). Transparency in interpretation and logical support for conclusions(C5). Identification of potential researcher influence or interpretive bias.

The resulting evaluations with overall risk classification (low, moderate, high) were consolidated into a risk-of-bias matrix, providing a transparent overview of methodological quality and potential bias sources across the body of evidence. This assessment informed the narrative synthesis and enhanced confidence in the validity and interpretability of the review findings.

Identifier	C1	C2	C3	C4	C5	Risk summary
Aborujilah et al. [2021]	Aligned	Appropriate	Appropriate	Appropriate	Appropriate	Low
Ahmed et al. [2024]	Partial	Minor concerns	Minor concerns	Minor concerns	Minor concerns	Moderate
Aleburu et al. [2024]	Aligned	Appropriate	Appropriate	Appropriate	Appropriate	Low
Ali Saberi et al. [2022]	Aligned	Minor concerns	Minor concerns	Appropriate	Minor concerns	Moderate
Amaracitra and Alamsyah [2025]	Aligned	Appropriate	Appropriate	Appropriate	Appropriate	Low
Anastasiadou et al. [2024]	Aligned	Appropriate	Appropriate	Appropriate	Appropriate	Low

Identifier	C1	C2	C3	C4	C5	Risk summary
Araujo et al. [2022]	Aligned	Appropriate	Appropriate	Appropriate	Appropriate	Low
Arsheen and Ahmad [2022]	Aligned	Appropriate	Appropriate	Appropriate	Appropriate	Low
Arunachalam et al. [2023]	Aligned	Appropriate	Appropriate	Appropriate	Appropriate	Low
Al-Aswad et al. [2021]	Aligned	Appropriate	Appropriate	Appropriate	Appropriate	Low
Bandi and Fellah [2022]	Partial	Appropriate	Minor concerns	Minor concerns	Minor concerns	Moderate
Bazel et al. [2022]	Partial	Appropriate	Minor concerns	Minor concerns	Minor concerns	Moderate
Benbrahim et al. [2024]	Aligned	Appropriate	Minor concerns	Minor concerns	Minor concerns	Moderate
Biswas et al. [2021]	Partial	Appropriate	Minor concerns	Minor concerns	Minor concerns	Moderate
Biswas et al. [2022]	Aligned	Appropriate	Appropriate	Appropriate	Appropriate	Low
Samonte et al. [2024]	Aligned	Appropriate	Appropriate	Appropriate	Appropriate	Low
Cheng et al. [2022]	Aligned	Appropriate	Appropriate	Appropriate	Appropriate	Low
Swami Das et al. [2023]	Partial	Appropriate	Minor concerns	Minor concerns	Minor concerns	Moderate
Debauche et al. [2022]	Aligned	Appropriate	Appropriate	Appropriate	Appropriate	Low
De Sousa and Coutinho [2022]	Aligned	Appropriate	Appropriate	Appropriate	Appropriate	Low
Diaz and Kaschel [2022]	Aligned	Appropriate	Appropriate	Appropriate	Appropriate	Low
Diaz et al. [2024]	Aligned	Appropriate	Appropriate	Appropriate	Appropriate	Low
Dowden et al. [2024]	Aligned	Appropriate	Appropriate	Appropriate	Appropriate	Low
Aryanto et al. [2021]	Aligned	Appropriate	Minor concerns	Minor concerns	Minor concerns	Moderate
Fragidis et al. [2024]	Aligned	Appropriate	Appropriate	Appropriate	Appropriate	Low
Gamal et al. [2021]	Aligned	Appropriate	Minor concerns	Minor concerns	Minor concerns	Moderate
S et al. [2024]	Aligned	Appropriate	Appropriate	Appropriate	Appropriate	Low
Gazzarata et al. [2021]	Aligned	Appropriate	Appropriate	Appropriate	Appropriate	Low

**Identifier**	**C1**	**C2**	**C3**	**C4**	**C5**	**Risk summary**
Guimaraes et al. [2023]	Aligned	Appropriate	Appropriate	Appropriate	Appropriate	Low
Haliima et al. [2022]	Aligned	Appropriate	Appropriate	Appropriate	Appropriate	Low
Haritha and Anitha [2023]	Aligned	Appropriate	Appropriate	Appropriate	Appropriate	Low
Hasselgren et al. [2021]	Aligned	Appropriate	Appropriate	Appropriate	Appropriate	Low
Hoque et al. [2024]	Aligned	Appropriate	Minor concerns	Minor concerns	Minor concerns	Moderate
Hu and Kar [2024]	Aligned	Appropriate	Appropriate	Appropriate	Appropriate	Low
Ivanov and Ivanov [2022]	Aligned	Appropriate	Minor concerns	Minor concerns	Minor concerns	Moderate
Kalita et al. [2023]	Aligned	Appropriate	Appropriate	Appropriate	Appropriate	Low
Kastowo et al. [2022]	Aligned	Appropriate	Appropriate	Appropriate	Appropriate	Low
Klementi et al. [2024]	Aligned	Appropriate	Appropriate	Appropriate	Appropriate	Low
Koren et al. [2022]	Aligned	Appropriate	Appropriate	Appropriate	Appropriate	Low
Koren and Prasad [2024]	Aligned	Appropriate	Appropriate	Appropriate	Appropriate	Low
Kumar et al. [2021]	Aligned	Appropriate	Appropriate	Appropriate	Appropriate	Low
Kumar et al. [2023]	Aligned	Appropriate	Appropriate	Appropriate	Appropriate	Low
Kumari et al. [2021]	Aligned	Appropriate	Minor concerns	Minor concerns	Minor concerns	Moderate
Kumhar et al. [2023]	Aligned	Appropriate	Appropriate	Appropriate	Appropriate	Low
Lee and Kim [2022]	Aligned	Appropriate	Minor concerns	Minor concerns	Minor concerns	Moderate
Lindgren et al. [2021]	Aligned	Appropriate	Appropriate	Appropriate	Appropriate	Low
Lindquist et al. [2021]	Aligned	Appropriate	Appropriate	Appropriate	Appropriate	Low
López-Úbeda et al. [2021]	Aligned	Appropriate	Appropriate	Appropriate	Appropriate	Low
Shravan et al. [2022]	Aligned	Appropriate	Appropriate	Appropriate	Appropriate	Low

Identifier	C1	C2	C3	C4	C5	Risk summary
Malik et al. [2024]	Aligned	Appropriate	Appropriate	Appropriate	Appropriate	Low
Mammadova and Ahmadova [2022]	Partial	Appropriate	Minor concerns	Minor concerns	Minor concerns	Moderate
Manoharan et al. [2024]	Aligned	Appropriate	Appropriate	Appropriate	Appropriate	Low
Marinescu et al. [2021]	Partial	Appropriate	Minor concerns	Minor concerns	Minor concerns	Moderate
Marques et al. [2022]	Aligned	Appropriate	Minor concerns	Minor concerns	Minor concerns	Moderate
Messai et al. [2024]	Aligned	Appropriate	Minor concerns	Minor concerns	Minor concerns	Moderate
Mishra and Prasad [2022]	Aligned	Appropriate	Appropriate	Appropriate	Appropriate	Low
Murat et al. [2023]	Aligned	Appropriate	Appropriate	Appropriate	Minor concerns	Low
Mutasa et al. [2025]	Aligned	Appropriate	Appropriate	Minor concerns	Appropriate	Low
Nabasirye and Ssali [2025]	Aligned	Appropriate	Appropriate	Appropriate	Appropriate	Low
Nazakat et al. [2022]	Aligned	Appropriate	Appropriate	Appropriate	Appropriate	Low
Olca and Can [2022]	Aligned	Appropriate	Appropriate	Appropriate	Appropriate	Low
Osei-Tutu and Song [2021]	Aligned	Appropriate	Appropriate	Appropriate	Minor concerns	Moderate
Perbix et al. [2022]	Aligned	Appropriate	Appropriate	Appropriate	Appropriate	Low
Piamjinda et al. [2024]	Aligned	Appropriate	Appropriate	Appropriate	Appropriate	Low
Qureshi et al. [2024]	Aligned	Appropriate	Appropriate	Appropriate	Appropriate	Low
Chaitra and Dhananjaya [2023]	Aligned	Appropriate	Appropriate	Appropriate	Appropriate	Low
Rajasekharan and Koshy [2024]	Aligned	Appropriate	Appropriate	Appropriate	Appropriate	Low
Ricci et al. [2022]	Aligned	Appropriate	Appropriate	Appropriate	Appropriate	Low
Rinty et al. [2022]	Aligned	Appropriate	Appropriate	Appropriate	Appropriate	Low
Rojo et al. [2023]	Aligned	Appropriate	Appropriate	Appropriate	Appropriate	Low
Rwegasira et al. [2024]	Aligned	Appropriate	Appropriate	Appropriate	Appropriate	Low

Identifier	C1	C2	C3	C4	C5	Risk summary
Snegireva et al. [2021]	Aligned	Appropriate	Appropriate	Appropriate	Appropriate	Low
Sundararaman [2023]	Aligned	Appropriate	Appropriate	Appropriate	Appropriate	Low
Surasak [2024]	Aligned	Appropriate	Appropriate	Appropriate	Appropriate	Low
Komal et al. [2022]	Aligned	Appropriate	Appropriate	Appropriate	Appropriate	Low
Thamrin and Xu [2021]	Partial	Minor concerns	Minor concerns	Minor concerns	Minor concerns	Moderate
He et al. [2022]	Partial	Minor concerns	Minor concerns	Minor concerns	Minor concerns	Moderate
Tsegaye and Flowerday [2021]	Aligned	Appropriate	Appropriate	Appropriate	Minor concerns	Low
Tummers et al. [2021]	Aligned	Appropriate	Appropriate	Appropriate	Minor concerns	Low
Tummers et al. [2024]	Aligned	Appropriate	Appropriate	Appropriate	Appropriate	Low
Vellela et al. [2023]	Partial	Minor concerns	Minor concerns	Minor concerns	Minor concerns	Moderate
Weng et al. [2021]	Partial	Minor concerns	Minor concerns	Minor concerns	Minor concerns	Moderate
Wijayanti et al. [2024]	Aligned	Appropriate	Appropriate	Appropriate	Minor concerns	Low
Winter et al. [2025]	Aligned	Appropriate	Appropriate	Appropriate	Minor concerns	Low
Yongjoh et al. [2021]	Aligned	Appropriate	Appropriate	Appropriate	Minor concerns	Low
Zampognaro et al. [2021]	Aligned	Appropriate	Minor concerns	Minor concerns	Minor concerns	Moderate
Zao et al. [2024]	Aligned	Appropriate	Appropriate	Appropriate	Minor concerns	Low
Zhang et al. [2022]	Partial	Minor concerns	Minor concerns	Minor concerns	Minor concerns	Moderate
Zhang et al. [2023]	Aligned	Appropriate	Appropriate	Appropriate	Minor concerns	Low

Table S3: Narrative risk-of-bias detailing methodological coherence and transparency of the 89 included studies. Each dimension reflects judgment criteria adapted from JBI tools and applied to qualitative and design-science evidence within digital-health architecture research.

REFERENCES

A. Aborujilah, A. E. F. M. Elsebaie, and S. A. Mokhtar. IoT MEMS: IoT-Based Paradigm for Medical Equipment Management Systems of ICUs in Light of COVID-19 Outbreak. *IEEE Access*, 9:131120–131133, 2021. ISSN 21693536. doi: 10.1109/ACCESS.2021.3069255.

K. R. Ahmed, R. Islam, M. A. Alam, M. A. H. Rivin, M. Alam, and M. S. Rahman. A Management Information Systems Framework for Sustainable Cloud-Based Smart E-Healthcare Research Information Systems in Bangladesh. In *2024 Asian Conference on Intelligent Technologies, ACOIT 2024*. Institute of Electrical and Electronics Engineers Inc., 2024. ISBN 9798350374933. doi: 10.1109/ACOIT62457.2024.10941651.

H. Al-Aswad, W. M. El-Medany, C. Balakrishna, N. Ababneh, and K. Curran. BZKP: Blockchain-based zero-knowledge proof model for enhancing healthcare security in Bahrain IoT smart cities and COVID-19 risk mitigation. *Arab Journal of Basic and Applied Sciences*, 28(1):154–171, 2021. ISSN 25765299. doi: 10.1080/25765299.2020.1870812.

D. Aleburu, C. Mabude, A. Mustapha, U. Umoren, and S. Kuyoro. HINE-Block: Blockchain based Model for Secure Health Information Exchange. In *International Conference on Science, Engineering and Business for Driving Sustainable Development Goals, SEB4SDG 2024*. Institute of Electrical and Electronics Engineers Inc., 2024. ISBN 9798350358155. doi: 10.1109/SEB4SDG60871.2024.10629816.

M. Ali Saberi, H. McHeick, M. Adda, and H. Ibrahim. Toward Implementing Interoperability in Pervasive Healthcare Systems for Chronic Diseases By Decentralization and Modularity. In *2022 3rd International Conference on Human-Centric Smart Environments for Health and Well-Being, IHSH 2022*, pages 64–72. Institute of Electrical and Electronics Engineers Inc., 2022. ISBN 9781665463218. doi: 10.1109/IHSH57076.2022.10092028.

D. S. Amaracitra and A. Alamsyah. Decentralized Medical Record Model Using Composable NFT: Achieving Data Security, Interoperability, and Privacy through Granular Data Access. pages 1–6. Institute of Electrical and Electronics Engineers (IEEE), 6 2025. ISBN 9798331519018. doi: 10.1109/iceeng64546.2025.11031352.

M. N. Anastasiadou, P. Isaia, P. Kolios, D. G. Eliades, and C. Laoudias. Leveraging ICTs for Effective COVID-19 Pandemics Management: Insights from a Health Information System Implementation in Cyprus. In *Proceedings - 2024 IEEE/ACM 24th International Symposium on Cluster, Cloud and Internet Computing Workshops, CCGridW 2024*, pages 84–91. Institute of Electrical and Electronics Engineers Inc., 2024. ISBN 9798350395662. doi: 10.1109/CCGridW63211.2024.00016.

A. Araujo, F. De Barros Vidal, A. C. F. Martins, R. Bonifacio, M. Atique, and J. H. C. Fernandes. An overview of the Information Systems in Primary Care of the Brazil's Unified Health System. In *2022 17th Iberian Conference on Information Systems and Technologies (CISTI)*, pages 1–6. IEEE, 6 2022. ISBN 978-9-8933-3436-2. doi: 10.23919/CISTI54924.2022.9820222. URL `https://ieeexplore.ieee.org/document/9820222/`.

S. Arsheen and K. Ahmad. Blockchain-Enabled Immunization System: A Novel Idea to Leverage Reliability and Traceability. In *2022 3rd International Conference for Emerging Technology, INCET 2022*. Institute of Electrical and Electronics Engineers Inc., 2022. ISBN 9781665494991. doi: 10.1109/INCET54531.2022.9824500.

S. Arunachalam, H. J. Shanthi, G. Sivagurunathan, S. Das, D. Anand, and T. Raj M. Cloud-based Decentralized Smart Healthcare for Patient Monitoring on Deep Learning. In *Proceedings of the 2nd International Conference on Applied Artificial Intelligence and Computing, ICAAIC 2023*, pages 459–466. Institute of Electrical and Electronics Engineers Inc., 2023. ISBN 9781665456302. doi: 10.1109/ICAAIC56838.2023.10141120.

K. Y. E. Aryanto, K. A. Seputra, I. N. S. W. Wijaya, I. W. Abyong, G. A. Pradnyana, and A. A. Y. Paramartha. Towards Healthcare Data Sharing: An e-Health Integration Effort in Indonesian District. In *Proceedings - 2021 International Seminar on Application for Technology of Information and Communication: IT Opportunities and Creativities for Digital Innovation and Communication within Global Pandemic, iSemantic 2021*, pages 280–284. Institute of Electrical and Electronics Engineers Inc., 9 2021. ISBN 9781665428040. doi: 10.1109/iSemantic52711.2021.9573250.

A. Bandi and A. Fellah. An Implementation and Evaluation of Blockchain-based Digital Health Passports. In *5th International Conference on Inventive Computation Technologies, ICICT 2022 - Proceedings*, pages 476–482. Institute of Electrical and Electronics Engineers Inc., 2022. ISBN 9781665408370. doi: 10.1109/ICICT54344.2022.9850724.

M. A. Bazel, M. Ahmad, and F. Mohammed. Hospital Information Systems in Malaysia: Current Issues and Blockchain Technology as a Solution. In *2022 2nd International Conference on Emerging Smart Technologies and Applications, eSmarTA 2022*. Institute of Electrical and Electronics Engineers Inc., 2022. ISBN 9781665461818. doi: 10.1109/eSmarTA56775.2022.9935387.

H. Benbrahim, H. Hachimi, and A. Amine. The Moroccan Health Data Bank: A Proposal for a National Electronic Health System Based on Big Data. *Journal of Information Technology Management*, 16(1): 79–97, 2024. ISSN 24235059. doi: 10.22059/jitm.2024.96376.

S. Biswas, K. Sharif, F. Li, A. K. Bairagi, Z. Latif, and S. P. Mohanty. GlobeChain: An Interoperable Blockchain for Global Sharing of Healthcare Data - A COVID-19 Perspective. *IEEE Consumer Electronics Magazine*, 10(5):64–69, 9 2021. ISSN 21622256. doi: 10.1109/MCE.2021.3074688.

S. Biswas, K. Sharif, F. Li, I. Alam, and S. P. Mohanty. DAAC: Digital asset access control in a unified blockchain based e-health system. *IEEE Transactions on Big Data*, 8(5):1273–1287, 10 2022. ISSN 23327790. doi: 10.1109/TBDATA.2020.3037914.

R. Chaitra and V. Dhananjaya. An Enhanced Mechanism for Securing Patient Vital Health Information in Public Cloud. In *Proceedings of the 2023 2nd International Conference on Augmented Intelligence and Sustainable Systems, ICAISS 2023*, pages 1323–1328. Institute of Electrical and Electronics Engineers Inc., 2023. ISBN 9798350325799. doi: 10.1109/ICAISS58487.2023.10250739.

D. Cheng, Y. Fu, and Y. Zhou. Basic Medical Information Sharing System Design Based on IoT Technology. In *Proceedings - 2022 International Conference on Intelligent Transportation, Big Data and Smart City, ICITBS 2022*, pages 375–378. Institute of Electrical and Electronics Engineers Inc., 2022. ISBN 9781665497213. doi: 10.1109/ICITBS55627.2022.00087.

O. V. J. De Sousa and C. Coutinho. Interoperability Between Information Systems Concerning Electronic Records of Patients. In *2022 International Symposium on Sensing and Instrumentation in 5G and IoT Era, ISSI 2022*, pages 121–126. Institute of Electrical and Electronics Engineers Inc., 2022. ISBN 9781665498289. doi: 10.1109/ISSI55442.2022.9963271.

O. Debauche, J. B. Nkamla Penka, S. Mahmoudi, X. Lessage, M. Hani, P. Manneback, U. K. Lufuluabu, N. Bert, D. Messaoudi, and A. Guttadauria. RAMi: A New Real-Time Internet of Medical Things Architecture for Elderly Patient Monitoring. *Information (Switzerland)*, 13(9), 9 2022. ISSN 20782489. doi: 10.3390/info13090423.

A. Diaz and H. Kaschel. Scalable Management Architecture for Electronic Health Records Based on Blockchain. In *2022 IEEE International Conference on Automation/25th Congress of the Chilean Association of Automatic Control: For the Development of Sustainable Agricultural Systems, ICA-ACCA 2022*. Institute of Electrical and Electronics Engineers Inc., 2022. ISBN 9781665494083. doi: 10.1109/ICA-ACCA56767.2022.10005960.

A. G. Diaz, A. D. Gumtang, C. J. A. Orpiada, A. S. Balagot, E. A. Villanueva, and M. A. Manalang. PHIrecord: A Medical Record Management System for Rural Health Facilities in the Philippines. In *2024 6th IEEE Symposium on Computers and Informatics, ISCI 2024*, pages 188–193. Institute of Electrical and Electronics Engineers Inc., 2024. ISBN 9798350353853. doi: 10.1109/ISCI62787.2024.10668022.

J. J. Dowden, R. W. Pretty, J. M. Shea, M. Dermody, G. Doyle, S. Antle, and D. Bond. A novel technology for harmonizing and analyzing cancer data. Observations from integrating health connect in Newfoundland and Labrador, Canada. *Health Informatics Journal*, 30(3), 7 2024. ISSN 17412811. doi: 10.1177/14604582241267792.

L. Fragidis, S. Tsamoglou, K. Kosmidis, and V. Aggelidis. Architectural design of national evidence based medicine information system based on electronic health record. *Technology and Health Care*, 32(6): 4187–4201, 11 2024. ISSN 09287329. doi: 10.3233/THC-232042.

A. Gamal, S. Barakat, and A. Rezk. Integrated Document-based Electronic Health Records Persistence Framework. *International Journal of Advanced Computer Science and Applications*, 12(10):2021, 2021. ISSN 21565570. doi: 10.14569/IJACSA.2021.0121017. URL `http://thesai.org/Publications/ViewPaper?Volume=12&Issue=10&Code=IJACSA&SerialNo=17`.

R. Gazzarata, N. Maggi, L. D. Magnoni, M. E. Monteverde, C. Ruggiero, and M. Giacomini. Semantics Management for a Regional Health Information System in Italy by CTS2 and FHIR. In *Studies in Health Technology and Informatics*, volume 287, pages 119–123. IOS Press BV, 11 2021. ISBN 9781643682365. doi: 10.3233/SHTI210828.

T. Guimaraes, R. Duarte, J. Cunha, P. Gomes, and M. F. Santos. Security and Immutability of Open Data in Healthcare. In *Procedia Computer Science*, volume 220, pages 832–837. Elsevier B.V., 2023. doi: 10.1016/j.procs.2023.03.111.

N. Haliima, G. Rushingabigwi, and F. Nzanywayingoma. Design of an IoT Based Monitoring System for Expectant Rural Women in Developing Countries. In *Proceedings of the 2nd 2022 International Conference on Computer Science and Software Engineering, CSASE 2022*, pages 41–47. Institute of Electrical and Electronics Engineers Inc., 2022. ISBN 9781665426329. doi: 10.1109/CSASE51777.2022.9759594.

T. Haritha and A. Anitha. Multi-Level Security in Healthcare by Integrating Lattice-Based Access Control and Blockchain- Based Smart Contracts System. *IEEE Access*, 11:114322–114340, 2023. ISSN 21693536. doi: 10.1109/ACCESS.2023.3324740.

A. Hasselgren, J. A. H. Rensaa, K. Kralevska, D. Gligoroski, and A. Faxvaag. Blockchain for increased trust in virtual health care: Proof-of-concept study. *Journal of Medical Internet Research*, 23(7), 7 2021. ISSN 14388871. doi: 10.2196/28496.

W. He, J. Z. Zhang, H. Wu, W. Li, and S. Shetty. A Unified Health Information System Framework for Connecting Data, People, Devices, and Systems. *Journal of Global Information Management*, 30(11), 2022. ISSN 15337995. doi: 10.4018/JGIM.305239.

A. S. M. L. Hoque, M. R. Mia, M. S. Abdullah, M. J. Islam, B. C. D. Nath, M. T. Rahman, and S. I. Ahamed. BlockPRLS: Blockchain-Based Patient Record Linkage System for Big Data Analytics. In *Proceedings - 2024 IEEE 48th Annual Computers, Software, and Applications Conference, COMPSAC 2024*, pages 877–886. Institute of Electrical and Electronics Engineers Inc., 2024. ISBN 9798350376968. doi: 10.1109/COMPSAC61105.2024.00121.

L. Hu and P. Kar. Design of a Blockchain-Based Secure Health Monitoring System Using Decentralized Machine Learning Technique. *IEEE Communications Magazine*, 62(1):46–52, 1 2024. ISSN 15581896. doi: 10.1109/MCOM.002.2300610.

I. E. Ivanov and B. Ivanov. Unified National Digital Framework for Exchange and Storage of Medical Image Information. In *2022 10th International Scientific Conference on Computer Science, COMSCI 2022 - Proceedings*. Institute of Electrical and Electronics Engineers Inc., 2022. ISBN 9781665497770. doi: 10.1109/COMSCI55378.2022.9912601.

K. P. Kalita, S. K. Chettri, and R. K. Deka. A Blockchain-based Model for Maternal Health Information Exchange and Prediction of Health Risks using Machine Learning. In *Proceedings of the International Conference on Intelligent and Innovative Technologies in Computing, Electrical and Electronics, ICIITCEE 2023*, pages 1184–1189. Institute of Electrical and Electronics Engineers Inc., 2023. ISBN 9781665462631. doi: 10.1109/IITCEE57236.2023.10090997.

D. Kastowo, E. Utami, and A. Hendi Muhammad. FHIR, BigchainDB, and GraphQL approach for interoperability between heterogeneous Health Information System. In *ICOIACT 2022 - 5th International Conference on Information and Communications Technology: A New Way to Make AI Useful for Everyone in the New Normal Era, Proceeding*, pages 272–277. Institute of Electrical and Electronics Engineers Inc., 2022. ISBN 9781665451406. doi: 10.1109/ICOIACT55506.2022.9972042.

T. Klementi, G. Piho, and P. Ross. A reference architecture for personal health data spaces using decentralized content-addressable storage networks. *Frontiers in Medicine*, 11, 2024. ISSN 2296858X. doi: 10.3389/fmed.2024.1411013.

T. Komal, S. Kandasamy, S. Meenalakshmi, and R. M. Devi. Blockchain based Effective Ledger and Decentralization in Healthcare System. In *6th International Conference on Electronics, Communication and Aerospace Technology, ICECA 2022 - Proceedings*, pages 736–742. Institute of Electrical and Electronics Engineers Inc., 2022. ISBN 9781665482714. doi: 10.1109/ICECA55336.2022.10009365.

A. Koren and R. Prasad. Setting Standards for Personal Health Data in the Age of 5G and 6G Networks. *Journal of ICT Standardization*, 12(1):47–70, 2024. ISSN 22460853. doi: 10.13052/jicts2245-800X.1213.

A. Koren, M. Jurcevic, and R. Prasad. Semantic Constraints Specification and Schematron-Based Validation for Internet of Medical Things- Data. *IEEE Access*, 10:65658–65670, 2022. ISSN 21693536. doi: 10.1109/ACCESS.2022.3182486.

J. Kumar, A. S. Ali, A. Kumar, and S. Kumar. Enhancing Patient Data Security Through Blockchain Adoption in Fiji's Healthcare System. In *Proceedings of 2023 International Conference on Sustainable Technology and Engineering, i-COSTE 2023*. Institute of Electrical and Electronics Engineers Inc., 2023. ISBN 9798350329711. doi: 10.1109/i-COSTE60462.2023.10500773.

V. Kumar, A. Sarkar, and A. Jana. Big Data and WebGIS for Formulating Health Care Policy in India. In *2021 IEEE Conference on Norbert Wiener in the 21st Century: Being Human in a Global Village, 21CW 2021*. Institute of Electrical and Electronics Engineers Inc., 7 2021. ISBN 9781728153841. doi: 10.1109/21CW48944.2021.9532567.

T. Kumari, R. Kumari, N. Devi, and B. Sharma. Personalized Healthcare Monitoring System. In *Proceedings of the 5th International Conference on Trends in Electronics and Informatics, ICOEI 2021*, pages 1088–1096. Institute of Electrical and Electronics Engineers Inc., 6 2021. ISBN 9781665415712. doi: 10.1109/ICOEI51242.2021.9453056.

M. Kumhar, J. Bhatia, N. K. Jadav, A. A. Padaria, R. Gupta, S. Tanwar, and J. J. Rodrigues. HEAL-SDN: Artificial Neural Network-based Secure Data Exchange Framework for SDN Controllers in Healthcare 4.0. In *2023 IEEE Globecom Workshops, GC Wkshps 2023*, pages 1832–1837. Institute of Electrical and Electronics Engineers Inc., 2023. ISBN 9798350370218. doi: 10.1109/GCWkshps58843.2023.10465150.

S. Lee and S. Kim. Blockchain as a Cyber Defense: Opportunities, Applications, and Challenges. *IEEE Access*, 10:2602–2618, 2022. ISSN 21693536. doi: 10.1109/ACCESS.2021.3136328.

H. Lindgren, T. Kampik, E. G. Rosero, M. Blusi, and J. C. Nieves. Argumentation-Based Health Information Systems: A Design Methodology. *IEEE Intelligent Systems*, 36(2):72–80, 3 2021. ISSN 19411294. doi: 10.1109/MIS.2020.3044944.

W. Lindquist, S. Helal, A. Khaled, and W. Hutchinson. IoTility: Architectural Requirements for Enabling Health IoT Ecosystems. *IEEE Transactions on Emerging Topics in Computing*, 9(3):1206–1218, 2021. ISSN 21686750. doi: 10.1109/TETC.2019.2957241.

P. López-Úbeda, M. Carlos Díaz-Galiano, L. Alfonso Ureña-López, and M. Teresa Martín-Valdivia. Pre-trained language models to extract information from radiological reports. Technical report, 2021. URL `http://ceur-ws.org`.

R. Malik, A. Ur-Rehaman, H. Razzaq, C. Bhatt, K. Kaushik, and I. U. Khan. Advancing Healthcare IoT: Blockchain and Federated Learning Integration for Enhanced Security and Insights. In *Proceedings of International Conference on Communication, Computer Sciences and Engineering, IC3SE 2024*, pages 308–314. Institute of Electrical and Electronics Engineers Inc., 2024. ISBN 9798350366846. doi: 10.1109/IC3SE62002.2024.10593078.

M. Mammadova and A. Ahmadova. Formation of Unified Digital Health Information Space in Healthcare 4.0 Environment and interoperability issues. In *16th IEEE International Conference on Application of Information and Communication Technologies, AICT 2022 - Proceedings*. Institute of Electrical and Electronics Engineers Inc., 2022. ISBN 9781665451628. doi: 10.1109/AICT55583.2022.10013605.

J. Manoharan, A. H. Ali, M. M. Aljohani, A. Soni, V. Gowrishankar, and S. Upadhyay. Experimental Possibilities of Decentralized Health Information System Interoperability Using Blockchain Technology. In *5th International Conference on Sustainable Communication Networks and Application, ICSCNA 2024 - Proceedings*, pages 432–438. Institute of Electrical and Electronics Engineers Inc., 2024. ISBN 9798331530013. doi: 10.1109/ICSCNA63714.2024.10863833.

I. A. Marinescu, C. M. Rotaru, D. Nicolau, and P. Krawiec. Challenges and perspectives for the development of a future ecosystem for elderly within pandemic. In *Proceedings - 2021 23rd International Conference on Control Systems and Computer Science Technologies, CSCS 2021*, pages 501–508. Institute of Electrical and Electronics Engineers Inc., 5 2021. ISBN 9781665439398. doi: 10.1109/CSCS52396.2021.00088.

C. Marques, V. Ramos, H. Peixoto, and J. Machado. Pervasive Monitoring System for Services and Servers in Healthcare Environment. In *Procedia Computer Science*, volume 201, pages 720–725. Elsevier B.V., 2022. doi: 10.1016/j.procs.2022.03.097.

A. Messai, A. Drif, A. Ouyahia, M. Guechi, M. Rais, L. Kaderali, and H. Cherifi. Transparent AI Models for Meningococcal Meningitis Diagnosis: Evaluating Interpretability and Performance Metrics. In *International IEEE Conference proceedings, IS*, number 2024. Institute of Electrical and Electronics Engineers Inc., 2024. ISBN 9798350350982. doi: 10.1109/IS61756.2024.10705255.

R. Mishra and R. Prasad. Towards Efficient and Secure Framework for Devices and Informatics for Internet of Medical Things. In *International Symposium on Wireless Personal Multimedia Communications, WPMC*, volume 2022-October, pages 459–463. IEEE Computer Society, 2022. ISBN 9781665473187. doi: 10.1109/WPMC55625.2022.10014830.

B. Murat, A. O. Uzer, S. Ketenci, S. Yasbek, and I. Korkmaz. A Symptom Evaluation System on Medical Diagnosis. In *TIPTEKNO 2023 - Medical Technologies Congress, Proceedings*. Institute of Electrical and Electronics Engineers Inc., 2023. ISBN 9798350328967. doi: 10.1109/TIPTEKNO59875.2023.10359191.

L. Mutasa, M. M. Ujakpa, W. Nyikana, I. Shaanika, and T. Iyamu. Application of Enterprise Architecture to Guide the Integration of Health Information Systems in Namibia. *Information Resources Management Journal*, 38(1):1–22, 2025. ISSN 15337979. doi: 10.4018/IRMJ.367274.

A. Nabasirye and I. W. Ssali. Integrating Natural Language Processing and Large Language Models Into DHIS2 to Improve Health Data Utilization. pages 47–52. Institute of Electrical and Electronics Engineers (IEEE), 6 2025. ISBN 9798331514280. doi: 10.1109/seigs66664.2025.00012.

M. Nazakat, F. Khalique, S. A. Khan, and N. Ahsan. Towards Data Driven Spatio-Temporal Threshold Identification Based on Cost Effective Public Health Information Management Framework. *IEEE Access*, 10:16634–16643, 2022. ISSN 21693536. doi: 10.1109/ACCESS.2022.3149349.

E. Olca and O. Can. DICON: A Domain-Independent Consent Management for Personal Data Protection. *IEEE Access*, 10:95479–95497, 2022. ISSN 21693536. doi: 10.1109/ACCESS.2022.3204970.

K. Osei-Tutu and Y. T. Song. A Microservices Enterprise Architecture for Healthcare Information Exchange (HIE) in Developing Countries. In *Proceedings - 2021 10th International Congress on Advanced Applied Informatics, IIAI-AAI 2021*, pages 762–767. Institute of Electrical and Electronics Engineers Inc., 2021. ISBN 9781665424202. doi: 10.1109/IIAI-AAI53430.2021.00134.

M. J. Page, J. McKenzie, P. Bossuyt, I. Boutron, T. Hoffmann, c. mulrow, L. Shamseer, J. Tetzlaff, E. Akl, S. E. Brennan, R. Chou, J. Glanville, J. Grimshaw, A. Hróbjartsson, M. M. Lalu, T. Li, E. Loder, E. Mayo-Wilson, S. McDonald, L. A. McGuinness, L. Stewart, J. Thomas, A. Tricco, V. A. Welch, P. Whiting, and D. Moher. The PRISMA 2020 statement: an updated guideline for reporting systematic reviews, 9 2020.

M. Perbix, M. Löbe, S. Stäubert, A. Anil Sinaci, M. Gencturk, M. Quintero, A. Martinez-Garcia, C. Alvarez-Romero, C. L. Parra-Calderon, and A. Winter. A Formal Model for the FAIR4Health Information Architecture. In *Studies in Health Technology and Informatics*, volume 295, pages 446–449. IOS Press BV, 2022. ISBN 9781643682907. doi: 10.3233/SHTI220761.

P. Piamjinda, C. Boonnag, P. Ittichaiwong, S. Rattanasonrerk, K. Veerakanjana, K. Duangchaemkarn, W. Limpornchitwilai, K. Thanontip, N. Asawalertsak, T. Kaewlee, and T. Wilaiprasitporn. CHIVID: A Rapid Deployment of Community and Home Isolation during COVID-19 Pandemics. *IEEE Journal of Translational Engineering in Health and Medicine*, 12:390–400, 2024. ISSN 21682372. doi: 10.1109/JTEHM.2024.3377258.

N. S. Qureshi, D. Midhun Chakkaravarthy, and N. Jamal. Domestic Intranet Cloud Design for Connecting District Health Information Systems for Capacity Development of Healthcare in Developing Countries. In *1st International Conference on Innovative Engineering Sciences and Technological Research, ICIESTR 2024 - Proceedings*. Institute of Electrical and Electronics Engineers Inc., 2024. ISBN 9798350348637. doi: 10.1109/ICIESTR60916.2024.10798252.

A. Rajasekharan and R. Koshy. EMRChain: Electronic Medical Records Management System using Blockchain. In *2024 IEEE International Conference on Blockchain and Distributed Systems Security, ICBDS 2024*. Institute of Electrical and Electronics Engineers Inc., 2024. ISBN 9798350354348. doi: 10.1109/ICBDS61829.2024.10837244.

A. Ricci, A. Croatti, and S. Montagna. Pervasive and Connected Digital Twins - A Vision for Digital Health. *IEEE Internet Computing*, 26(5):26–32, 2022. ISSN 19410131. doi: 10.1109/MIC.2021.3052039.

M. R. Rinty, U. K. Prodhan, and M. M. Rahman. A prospective interoperable distributed e-Health system with loose coupling in improving healthcare services for developing countries. *Array*, 13, 3 2022. ISSN 25900056. doi: 10.1016/j.array.2021.100114.

J. Rojo, J. Garcia-Alonso, J. Berrocal, L. Foschini, P. Bellavista, J. Hernandez, and J. M. Murillo. Blockchains' federation: Developing Personal Health Trajectory-centered health systems. In *Proceedings*

- 23rd IEEE/ACM International Symposium on Cluster, Cloud and Internet Computing Workshops, CCGridW 2023, pages 81–88. Institute of Electrical and Electronics Engineers Inc., 2023. ISBN 9798350302080. doi: 10.1109/CCGridW59191.2023.00027.

D. Rwegasira, H. Kimaro, R. Mbiaji, J. Julius, V. Minde, B. Mussa, H. Kalist, D. Kibahila, M. Masembo, and A. Msasu. Deployment and Innovation Processes of Integrated Electronic Medical Record (EMR) System: A Case of University Health Centre Living Lab in Tanzania. In *2024 IST-Africa Conference (IST-Africa)*, pages 1–8. IEEE, 5 2024. ISBN 978-1-905824-73-1. doi: 10.23919/IST-Africa63983.2024.10569206.

G. S, A. J. Mabel Rani, A. P. S, C. Ravi, M. Jeyalaxmi, and V. S. Pandi. A Novel Approach to Design Secured and Privacy Enabled Health Data Linkage System based on Cyber Security Principles. In *2024 IEEE International Conference on Big Data & Machine Learning (ICBDML)*, pages 14–20. IEEE, 2 2024. ISBN 979-8-3503-7410-0. doi: 10.1109/ICBDML60909.2024.10577373.

M. J. C. Samonte, F. D. C. Magno, K. G. Y. Ang, and K. E. M. Lalap. AidConnect: A Telehealth Web-based System with Smartwatch Integration for Enhanced Medical Tracking Support in Aged Care Facilities for Caregivers of Elderly Individuals in the Philippines. In *Proceedings - 2024 13th International Conference on Computer Technologies and Development, TechDev 2024*, pages 96–102. Institute of Electrical and Electronics Engineers Inc., 2024. ISBN 9798331539771. doi: 10.1109/TechDev64369.2024.00025.

M. Shravan, H. A. Sanjay, K. A. Shastry, D. Rithesh Ramesh, V. Hemant, and K. Laxman. Interoperability in Blockchain based Healthcare. In *MysuruCon 2022 - 2022 IEEE 2nd Mysore Sub Section International Conference*. Institute of Electrical and Electronics Engineers Inc., 2022. ISBN 9781665497909. doi: 10.1109/MysuruCon55714.2022.9972496.

E. Snegireva, G. R. Khazankin, and I. Mikheenko. System Architecture for Reading and Interpreting Physical Printouts of Medical Forms. In *International Conference of Young Specialists on Micro/Nanotechnologies and Electron Devices, EDM*, volume 2021-June, pages 547–550. IEEE Computer Society, 6 2021. ISBN 9781665414982. doi: 10.1109/EDM52169.2021.9507594.

A. Sundararaman. Data Platform To Accelerate Healthcare Insights Generation. In *Proceedings - 2023 IEEE 11th International Conference on Healthcare Informatics, ICHI 2023*, pages 470–475. Institute of Electrical and Electronics Engineers Inc., 2023. ISBN 9798350302639. doi: 10.1109/ICHI57859.2023.00069.

T. Surasak. Blockchain-Enhanced Security and Efficiency for Thailand's Health Information System. Technical Report 11, 2024. URL `www.ijacsa.thesai.org`.

M. Swami Das, G. R. Kumar, and D. V. Lakshmi. Healthcare Mobile App for Rural Areas and Recent Advancements. In *5th IEEE International Conference on Cybernetics, Cognition and Machine Learning Applications, ICCCMLA 2023*, pages 209–220. Institute of Electrical and Electronics Engineers Inc., 2023. ISBN 9798350338287. doi: 10.1109/ICCCMLA58983.2023.10346913.

A. Thamrin and H. Xu. Hierarchical Cloud-Based Consortium Blockchains for Healthcare Data Storage. In *Proceedings - 2021 21st International Conference on Software Quality, Reliability and Security Companion, QRS-C 2021*, pages 644–651. Institute of Electrical and Electronics Engineers Inc., 2021. ISBN 9781665478366. doi: 10.1109/QRS-C55045.2021.00098.

T. Tsegaye and S. Flowerday. A System Architecture For Ensuring Interoperability In A South African National Electronic Health Record System. *South African Computer Journal*, 33(1):79–110, 7 2021. ISSN 23137835. doi: 10.18489/sacj.v33i1.838.

J. Tummers, H. Tobi, C. Catal, and B. Tekinerdogan. Designing a reference architecture for health information systems. *BMC Medical Informatics and Decision Making*, 21(1), 12 2021. ISSN 14726947. doi: 10.1186/s12911-021-01570-2.

J. Tummers, H. Tobi, C. Catal, B. Tekinerdogan, B. Schalk, and G. Leusink. A health information systems architecture study in intellectual disability care: Commonalities and variabilities. *Healthcare Analytics*, 5, 6 2024. ISSN 27724425. doi: 10.1016/j.health.2023.100295.

S. S. Vellela, V. L. Reddy, D. Roja, G. R. Rao, S. K. Khader Basha, and K. K. Kumar. A Cloud-Based Smart IoT Platform for Personalized Healthcare Data Gathering and Monitoring System. In *2023 3rd Asian Conference on Innovation in Technology, ASIANCON 2023*. Institute of Electrical and Electronics Engineers Inc., 2023. ISBN 9798350302288. doi: 10.1109/ASIANCON58793.2023.10270407.

X. Weng, H. Wu, Y. Pan, and H. Chen. Decentralized Personal Cloud Data Model and its Application in Campus Health Information System. In *2021 IEEE Intl Conf on Dependable, Autonomic and Secure Computing, Intl Conf on Pervasive Intelligence and Computing, Intl Conf on Cloud and Big Data Computing, Intl Conf on Cyber Science and Technology Congress (DASC/PiCom/CBDCom/CyberSciTech)*, pages 879–883. IEEE, 10 2021. ISBN 978-1-6654-2174-4. doi: 10.1109/DASC-PICom-CBDCom-CyberSciTech52372.2021.00146. URL `https://ieeexplore.ieee.org/document/9730502/`.

D. Wijayanti, S. Urbaya, T. Sitompul, and V. Adrian. E-Government Interoperability: Provincial-Level Architecture Model to Enable Fast Healthcare Interoperability Resources (FHIR). In *Proceedings of 2024 International Conference on Information Management and Technology, ICIMTech 2024*, pages 811–816. Institute of Electrical and Electronics Engineers Inc., 2024. ISBN 9798350390025. doi: 10.1109/ICIMTech63123.2024.10780873.

A. Winter, F. Jahn, M. Löbe, and S. Stäubert. The European Health Data Space as 3LGM2-Based Enterprise Architecture Model. *Studies in health technology and informatics*, 327:647–651, 5 2025. ISSN 18798365. doi: 10.3233/SHTI250428.

S. Yongjoh, C. So-In, P. Kompunt, P. Muneesawang, and R. I. Morien. Development of an Internet-of-Healthcare System Using Blockchain. *IEEE Access*, 9:113017–113031, 2021. ISSN 21693536. doi: 10.1109/ACCESS.2021.3103443.

P. Zampognaro, G. Paragliola, and V. Falanga. A FHIR based architecture of a multiprotocol IoT Home Gateway supporting dynamic plug of new devices within instrumented environments. In *Proceedings - IEEE Symposium on Computers and Communications*, volume 2021-September. Institute of Electrical and Electronics Engineers Inc., 2021. ISBN 9781665427449. doi: 10.1109/ISCC53001.2021.9631446.

J. K.-K. Zao, J. T.-S. Wu, K. Kanyimbo, F. Delizy, T.-T. Gan, H.-I. Kuo, C.-H. Hsia, C.-H. Lo, S.-H. Yang, C. J. A. Richard, B. Rajab, M. Monawe, B. Kamanga, N. Mtambalika, K.-L. J. Yu, C.-F. Chou, C.-A. Neoh, J. Gallagher, J. O'Donoghue, R. Mtegha, H.-Y. Lee, and A. Mbewe. Design of a Trustworthy Cloud-Native National Digital Health Information Infrastructure for Secure Data Management and Use. *Oxford Open Digital Health*, 2, 1 2024. doi: 10.1093/oodh/oqae043.

J. Zhang, J. Symons, P. Agapow, J. T. Teo, C. A. Paxton, J. Abdi, H. Mattie, C. Davie, A. Z. Torres, A. Folarin, H. Sood, L. A. Celi, J. Halamka, S. Eapen, and S. Budhdeo. Best practices in the real-world data life cycle. *PLOS Digital Health*, 1(1 January), 1 2022. ISSN 27673170. doi: 10.1371/journal.pdig.0000003.

K. Zhang, Q. Gao, J. Zhang, D. Dai, Y. Han, H. Zan, and X. Liu. Construction of Chinese Pediatric Epilepsy Knowledge Graph. In *2023 IEEE 36th International Symposium on Computer-Based Medical Systems (CBMS)*, pages 241–244. IEEE, 6 2023. ISBN 979-8-3503-1224-9. doi: 10.1109/CBMS58004.2023.00224.
